# Analysis of Cellular Heterogeneity in Immune Microenvironment of Primary Central Nervous System Lymphoma by Single-Cell Sequencing

**DOI:** 10.3389/fonc.2021.683007

**Published:** 2021-10-04

**Authors:** Boyuan Wei, Zhe Liu, Yue Fan, Shuwei Wang, Chao Dong, Wei Rao, Fan Yang, Gang Cheng, Jianning Zhang

**Affiliations:** ^1^ Department of Neurosurgery, The First Medical Center of People’s Liberation Army General (PLA) Hospital, Beijing, China; ^2^ Department of Computer Science, City University of Hong Kong, Hong Kong, Hong Kong, SAR China; ^3^ School of Public Health, Health Science Center of Xi’an Jiaotong University, Xi’an, China

**Keywords:** primary central nervous system lymphoma (PCNSL), single cell sequencing, cellular heterogeneity, immune microenvironment, pathogenic and therapeutic, cell communication

## Abstract

**Background:**

Primary central nervous system lymphoma (PCNSL) is characterized by a lack of specificity and poor prognosis. Further understanding of the tumor heterogeneity and molecular phenotype of PCNSL is of great significance for improving the diagnosis and treatment of this disease.

**Methods:**

To explore the distinct phenotypic states of PCNSL, transcriptome-wide single-cell RNA sequencing was performed on 34,851 PCNSL cells from patients. The cell types, heterogeneity, and gene subset enrichment of PCNSL were identified. A comparison of the PCNSL cells with 21,250 normal human fetal brain (nHFB) cells was further analyzed to reveal the differences between PCNSL and normal sample.

**Results:**

Six cell populations were mainly identified in the PCNSL tissue, including four types of immune cells—B cell, T cell, macrophage and dendritic cell—and two types of stromal cells: oligodendrocyte and meningeal cell. There are significant cellular interactions between B cells and several other cells. Three subpopulations of B cells indicating diffident functions were identified, as well as a small number of plasma cells. Different subtypes of T cells and dendritic cells also showed significant heterogeneity. It should be noted that, compared with normal, the gene expression and immune function of macrophages in PCNSL were significantly downregulated, which may be another important feature of PCNSL in addition to B cell lesions and may be a potential target for PCNSL therapy.

## Introduction

Primary central nervous system (CNS) lymphoma (PCNSL), defined as diffuse large B cell lymphoma (DLBCL) confined to the CNS, can occur in the setting of immunosuppression (HIV/AIDS, post-transplant) or in immunocompetent individuals (1, 2). The incidence of PCNSL accounting for 2% to 3% of all CNS neoplasms (3) has reportedly increased in the immunocompetent population (4). Incidence of PCNSL is recently increasing in the elderly (5). It is not possible to morphologically distinguish PCNSL and peripheral DLBCL. This non-Hodgkin aggressive B-cell lymphoma is distinguished from extra-cerebral DLBCL by its poorer prognosis. The tumor cells are mainly large, with numerous apoptotic cells or widespread necrosis, which commonly hinders diagnosis in small biopsies (6, 7).

The molecular subtype of PCNSL has been studied by different methodological approaches with conflicting conclusions. On the basis of several IHC studies, an ABC-like immunophenotype is typical (8, 9), but immunoglobulin heavy-chain gene mutational signatures also provide evidence for germinal center exposure, indicating that PCNSL develops from a B-cell that has been exposed to a germinal center influence outside the CNS (10–12). In addition, results of immunophenotyping studies suggest that tumor cells originate from a late germinal center to an early postgerminal center stage (13), while gene expression profiling studies indicate that PCNSLs are distributed among the spectrum of systemic DLBCL with roughly equal proportion of ABC and GC cases (14, 15).

Standard chemotherapeutic regimens for systemic DLBCL show little efficacy in PCNSL, likely because of inefficient drug delivery across the blood–brain barrier (16). High-dose methotrexate-based chemotherapy is the standard therapy for PCNSL. Chemotherapy with whole-brain radiation therapy has produced response rates up to 80%–90% and median overall survival up to 5 years. While treatment response rates are high, relapses are frequent and prognosis after recurrence is poor with 5-year survival rates ranging from 22% to 40% (17, 18). Up to 50% of patients with PCNSL will relapse, and 10%–15% demonstrate primary refractory disease, indicating a significant unmet therapeutic need (19). However, PCNSL has a poor prognosis compared with that of DLBCL (20) and the reason for the difference in prognosis between PCNSL and DLBCL has not been elucidated.

The rarity of the disease and the difficulty of obtaining intracranial specimens have hindered understanding of the pathophysiology of PCNSL. The observed overexpression of BCL6 and aberrant somatic hypermutation (aSHM) of many genes, together with expression of immunoglobulin M at the cell surface, have suggested that PCNSL cells may be arrested at the stage of terminal B-cell differentiation (21). The genomic alterations (GAs) underlying PCNSL have not been comprehensively studied. Single-nucleotide mutations in various genes, including MYD88, CD79b, PIM1, and BTG2, have been reported as the most prevalent genetic alterations in PCNSL (22–24).

Genome-wide gene expression in PCNSL compared with non-CNS DLBCL suggests that PCNSL has specific signatures to be distinguished from non-CNS DLBCL and greater molecular heterogeneity (14, 25). Of the genetic changes that lead to PCNSL, very little is known and no characteristic genetic alterations have been defined thus far. However, few retrospective studies have examined the detailed molecular network and cell signaling based on diagnosis with gene mutations and CNVs or the prognosis of patients with PCNSL.

Some vague evidences suggest that CNS lymphoma and peripheral lymphoma are heterogeneous, which may be related to different immune environments and different origins of lymphoma cells. At present, there is no clinical significance to reveal the molecular characteristics of CNS lymphoma.

Here, we performed an analysis of scRNA-seq data of CNS tissue sequenced by 10x Genomics to identify the cell types of CNS cells; we next performed differential expression (DE) analysis on two PCNSL-associated cells (i.e., B cell, plasma cell) and gene set enrichment analysis (GSEA) of their DE genes on the cell type-specific marker genes to examine the cell-specific functionalities in PCNSL development.

## Materials and Methods

### Single-Cell RNA Sequencing

Human PCNSL tumor samples were obtained surgically from clinical patients confirmed by pathology from our hospital. After sample collection processing and suspensions, we performed scRNA-seq following the manufacturer’s protocol. Briefly, the cells were washed with PBS and resuspended in 500 μl PBS. scRNA-seq libraries were prepared using a Chromium Single cell 3′ Reagent kit, version 2. Amplified cDNA and final libraries were evaluated using a High Sensitivity DNA Kit (Agilent Technologies). Sequencing was performed on NovaSeq 6000 (Illumina) at a depth of approximately 400M reads.

### Single-Cell Data Processing

The Cell Ranger software pipeline (version 4.0, http://support.10Xgenomics.com/single-cell-gene-expression/software/overview/welcome) provided by 10x Genomics was used to demultiplex cellular barcodes. Unique molecular identifier (UMI) counts were obtained by mapping reads to the human reference genome (GRCH38 3.1.0) genome and align transcriptomes using the STAR aligner and down-sample reads as required to generate normalized aggregate data across samples. In the end, a matrix of gene counts by cells was produced. We processed the UMI count matrix using the Seurat R package (version 4.0.2), resulting in 34,851 cells with 36,601 genes for PCNSL samples.

We first removed the likely multiplet captures, which is a major concern in microdroplet-base experiments through DoubletFinder. We filtered cells at the cell and gene levels to obtain the reliability results of PCNSL scRNA-seq data, respectively. We removed the low-quality cells with the following criteria: (i) the number of expressed genes was <200 or >2,000; (ii) the number of total counts was > 20,000; and (iii) the percentage of mitochondrial counts > 10%. We only kept the genes detected in at least 20 cells. After applying these quality control criteria, we obtained 20,307 cells with 12,229 genes in total, which were used for downstream analysis.

To analyze the differences between PCNSL and normal brain cells, we referred to a published scRNA-seq data of normal human fetal brain (nHFB). The raw data are from the European Genome-Phenome Archive: EGAS00001004422 (https://ega-archive.org/studies/EGAS00001004422). For accessibility reasons, we directly used the cellranger matrices from HFA567_total.filtered_gene_matrices, HFA570_total.filtered_gene_matrices, and HFA571_total.filtered_gene_matrices (https://datahub-262-c54.p.genap.ca/GBM_paper_data/GBM_cellranger_matrix.tar.gz) (26). The following criteria were used for nHFB scRNA data processing: (i) the number of expressed genes was <100 or >3,000; (ii) the number of total counts was > 20,000; and (iii) the percentage of mitochondrial counts > 10%. We only kept the genes detected in at least 20 cells. After applying these quality control criteria, we obtained 20,015 cells with 16,608 genes from 21,255 cells with 33,694 genes in total, which were used for downstream analysis.

### Cell Subpopulation Detection

scRNA-seq data were normalized using log transforms and scaled so that the mean expression across cells is 0 and the variance across cells is 1. Then, we performed dimension reduction and visualization on the single-cell data through the Seurat R package (version 4.0.2) (1, 27). We used the VST method (28) to obtain the top 3,000 highly variable genes and used the PCA method to reduce dimensionality. The top 21 principal components (PCs) were selected for tSNE to visualize the cell clustering (29). We then clustered the cells using the Leiden algorithm (30) implemented in the Seurat package, with the resolution parameter set to be 0.7. There are two methods for annotation cell types: (i) the Wilcox test was used to identify significantly differentially expressed genes (DEGs) between clusters. To identify the marker genes of each cluster, we combined the rest of the cells and set them to control cells. For the process of identifying DEGs between PCNSL and normal samples, we set cells of one cluster in PCNSL samples as the treatment group and the cells of this cluster in normal samples as the control group. We considered the genes with adjusted p-value < 0.05 and |log2FC|> 0.58 for each scRNA-seq cell type as the DEGs. The top 30 genes are used for the annotation cell type manual. According to the detailed intracellular marker gene, we combined with the CellMarker (http://bio-bigdata.hrbmu.edu.cn/CellMarker/) database. (ii) By comparing scRNA-seq with HumanPrimaryCellAtlasData and BlueprintEncodeData data, we utilized both the SingleR R package (version 1.4.1) and manual work to assign cell types to cluster automatic annotation. Finally, we compare the annotation results and determine the cell types.

### Gene Function Enrichment Analysis

We then performed gene ontology (GO) enrichment analysis using the database for annotation, visualization, and integrated discovery database (DAVID: version 6.8, with the significant DEGs https://david.ncifcrf.gov/summary.jsp). It is an essential tool for systematically extracting biological information from a set of genes. Adj. p-value < 0.05 was considered statistically significant.

### Cell Communication Analysis

In order to concretely prove the direct interaction between cell subpopulations, we used CellPhoneDB (version 2.1.7) to conduct interactive analysis on cell subpopulations. CellPhoneDB is a publicly available repository of selected receptors, ligands, and their interactions (31). Here, we used it (version 2.1.7) to explore the cell–cell communication by immune-related proteins. Here, we focus on the four classes of immune cell subpopulations and immune-related genes. There are eight classes of gene lists collected in our research: chemokines, T helper type 1 genes (Th1), T helper type 2 genes (Th2), T helper 17 genes (Th17), T regulators (Treg), and costimulatory, coinhibitory, and immune niche genes. The circle edge width is proportional to the number of cells in each cell cluster and the communication score between interacting cell clusters, respectively.

## Results

### Identification of Cell Types in PCNSL and nHFB

**Figure 1:**
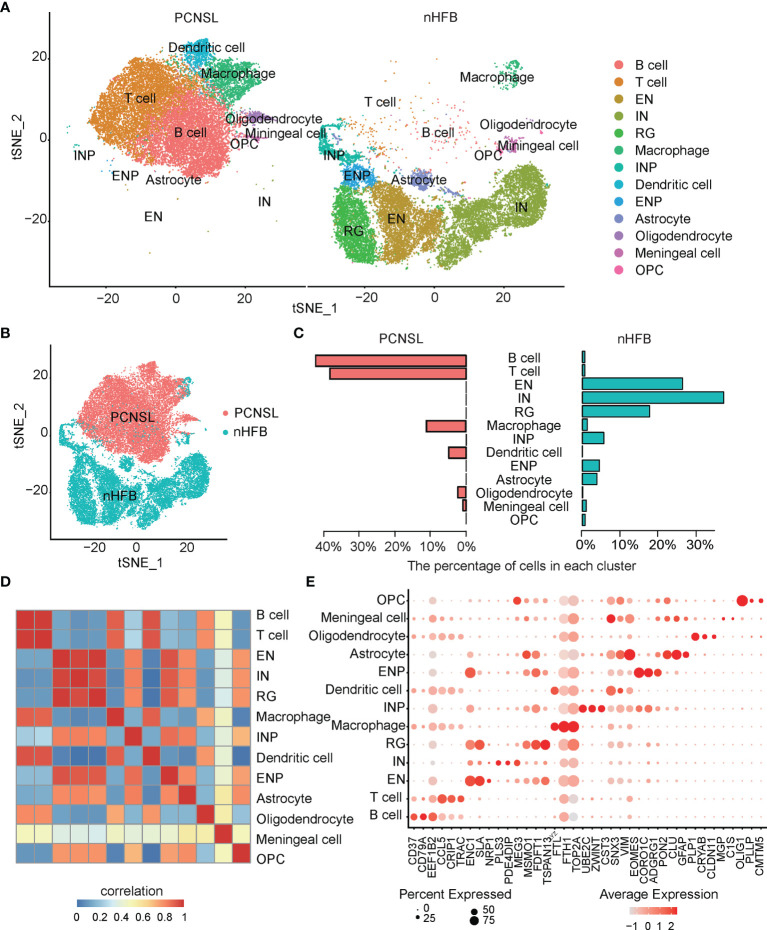
Single-cell analysis of CNS scRNA-seq data sets. **(A)** The visualization results of cell subpopulations in PCNSL and normal CNS tissue samples. **(B)** The tSNE displays PCNSL and normal CNS cells: red indicates PCNSL cells, and green indicates normal cells. **(C)** The barplot represents the proportion of various cell subgroups in PCNSL and normal CNS tissue samples individually. **(D)** The heatmap describes the PCC values of the average expression values of the top 30 genes in each cluster. **(E)** The bubble chart shows that three marker genes are selected for each cell subgroup, and their expression in all cell subpopulations is plotted.

After quality filtering, 40,322 cells with 20,452 genes were obtained from the PCNSL and nHFB samples (details in Materials and Methods). All cells were classified into 13 subpopulations by combining the automatic and manual annotation methods. The cell types were identified as B cell, T cell, macrophage, dendritic cell, oligodendrocyte, meningeal cell, excitatory neuron (EN), interneuron (IN), radial glia (RG), inhibitory neuronal progenitor cell (INP), excitatory neuronal progenitor (ENP), astrocyte, and oligodendrocyte progenitor cell (OPC) (**Figure 1A** and **Table 1**). The combination comparison (**Figures 1A, B**) indicated that PCNSL and nHFB had significant differences in cell heterogeneity, and there were certain similarities only in macrophages and meningeal cells. The B cell, T cell, macrophage, and dendritic cell population accounted for the highest proportion in PCNSL (**Table 2**, **Figure 1C**). By extracting the average value of top 30 gene expression in each group, and performing Pearson correlation coefficient (PCC) expression correlation analysis, the difference between groups is greater than the difference within the groups, which proves that our subpopulation annotation has high sensitivity and specificity (**Figure 1D**). The result of the bubble plot reveals that typical marker genes are particularly expressed in the cell subpopulations (**Figure 1E**). Overall, the annotation results show the complex cell composition in PCNSL tissue.

**Table 1:** The top30 marker genes in scRNA-seq data.

series no.	p_val	avg_log2FC	pct.1	pct.2	p_val_adj	cluster	gene
1	0	0.637447	0.18	0.658	0	B_cell	PDLIM1
2	0	0.631036	0.233	0.692	0	B_cell	CHCHD10
3	0	0.612104	0.128	0.648	0	B_cell	SH3TC1
4	0	0.575307	0.996	0.995	0	B_cell	RPS2
5	0	0.574816	0.304	0.757	0	B_cell	LTB
6	0	0.552174	0.927	0.946	0	B_cell	RPL5
7	0	0.537386	0.233	0.725	0	B_cell	CD53
8	0	0.524888	0.089	0.567	0	B_cell	S100A13
9	0	0.521512	0.181	0.698	0	B_cell	RGS10
10	0	0.519335	0.145	0.656	0	B_cell	POU2F2
11	0	0.516538	0.103	0.619	0	B_cell	MGST1
12	0	0.515477	0.139	0.644	0	B_cell	MTHFD2
13	0	0.514764	0.995	0.995	0	B_cell	RPS16
14	0	0.502641	0.912	0.948	0	B_cell	RPL7A
15	0	0.502393	0.981	0.988	0	B_cell	RPL12
16	6.43E-267	0.501824	0.849	0.913	1.29E-263	B_cell	RPS20
17	1.10E-249	0.634266	0.354	0.43	2.19E-246	B_cell	NCL
18	2.27E-243	0.804043	0.3	0.704	4.55E-240	B_cell	CD79A
19	1.99E-206	0.738747	0.296	0.689	3.97E-203	B_cell	GRHPR
20	3.12E-146	0.511718	0.623	0.733	6.24E-143	B_cell	HSP90AB1
21	1.37E-108	0.51492	0.717	0.864	2.74E-105	B_cell	EEF1B2
22	1.09E-55	0.569554	0.482	0.66	2.18E-52	B_cell	TPI1
23	1.56E-44	0.546826	0.597	0.81	3.11E-41	B_cell	NAP1L1
24	2.28E-42	0.518154	0.437	0.8	4.55E-39	B_cell	ARPC1B
25	9.31E-34	0.509425	0.429	0.623	1.86E-30	B_cell	ENO1
26	3.17E-24	0.502253	0.367	0.571	6.34E-21	B_cell	PRDX1
27	1.71E-15	0.582639	0.558	0.817	3.42E-12	B_cell	NME2
28	2.95E-14	0.532131	0.464	0.701	5.90E-11	B_cell	HNRNPA2B1
29	4.09E-07	0.778102	0.451	0.767	0.000818	B_cell	CD37
30	2.90E-05	0.741066	0.468	0.786	0.057983	B_cell	LAPTM5
31	0	1.207199	0.783	0.849	0	T_cell	CCL5
32	0	1.032656	0.779	0.854	0	T_cell	S100A4
33	0	1.008933	0.474	0.449	0	T_cell	VIM
34	0	0.918082	0.261	0.68	0	T_cell	ITGB2
35	0	0.910773	0.229	0.667	0	T_cell	IL7R
36	5.79E-285	1.19201	0.658	0.796	1.16E-281	T_cell	CRIP1
37	3.44E-243	0.867936	0.292	0.699	6.88E-240	T_cell	CD53
38	3.20E-232	1.150913	0.645	0.791	6.41E-229	T_cell	RGS1
39	1.63E-199	1.087939	0.637	0.799	3.27E-196	T_cell	SRGN
40	1.02E-193	0.867767	0.323	0.714	2.05E-190	T_cell	NEAT1
41	2.29E-193	1.141927	0.294	0.677	4.58E-190	T_cell	ITM2A
42	4.65E-155	1.01862	0.63	0.794	9.29E-152	T_cell	CCL4
43	1.28E-153	0.891872	0.332	0.701	2.56E-150	T_cell	PRDM1
44	4.56E-148	0.919638	0.332	0.706	9.11E-145	T_cell	CD69
45	1.32E-133	0.875134	0.336	0.703	2.63E-130	T_cell	IFITM2
46	4.80E-128	1.079405	0.626	0.795	9.60E-125	T_cell	HMGB2
47	1.47E-115	0.917095	0.345	0.709	2.95E-112	T_cell	RAC2
48	2.11E-100	1.079114	0.343	0.694	4.21E-97	T_cell	TRBC1
49	2.47E-96	0.999457	0.353	0.702	4.95E-93	T_cell	DUSP2
50	3.97E-89	1.099723	0.364	0.715	7.93E-86	T_cell	CCL4L2
51	8.47E-87	0.925325	0.373	0.729	1.69E-83	T_cell	LTB
52	9.13E-64	1.1652	0.549	0.761	1.83E-60	T_cell	TRAC
53	1.10E-58	1.102035	0.369	0.699	2.20E-55	T_cell	TRBC2
54	1.25E-42	0.948514	0.4	0.729	2.50E-39	T_cell	PTPRC
55	5.60E-40	0.956439	0.551	0.775	1.12E-36	T_cell	S100A6
56	4.81E-32	1.013335	0.533	0.751	9.62E-29	T_cell	ZFP36L2
57	5.08E-29	1.011943	0.402	0.713	1.02E-25	T_cell	LGALS1
58	8.52E-16	0.996155	0.518	0.764	1.70E-12	T_cell	HLA-E
59	7.12E-14	0.974021	0.515	0.759	1.42E-10	T_cell	HCST
60	0.001593	1.060656	0.44	0.72	1	T_cell	FXYD2
61	0	1.356559	0.894	0.232	0	EN	ENC1
62	0	0.750511	0.743	0.272	0	EN	EGR1
63	0	0.655106	0.835	0.284	0	EN	SLA
64	0	0.599588	0.857	0.383	0	EN	JUN
65	0	0.551619	0.273	0.043	0	EN	NRP1
66	0	0.483026	0.905	0.309	0	EN	BASP1
67	0	0.481532	0.807	0.271	0	EN	SDCBP
68	0	0.452978	0.799	0.292	0	EN	EIF1B
69	0	0.421184	0.898	0.423	0	EN	FOS
70	0	0.407631	0.738	0.216	0	EN	MIAT
71	0	0.360442	0.285	0.063	0	EN	COL9A2
72	3.82E-183	0.35539	0.695	0.21	7.64E-180	EN	APLP1
73	1.89E-128	0.508965	0.651	0.193	3.79E-125	EN	STMN4
74	1.74E-119	0.390235	0.612	0.165	3.47E-116	EN	MDK
75	5.25E-113	0.631659	0.387	0.074	1.05E-109	EN	EPHB6
76	2.18E-112	0.382722	0.399	0.08	4.37E-109	EN	EEF1A2
77	9.93E-107	0.367838	0.274	0.076	1.99E-103	EN	KLHL23
78	4.55E-73	0.326634	0.344	0.114	9.09E-70	EN	MED19
79	1.36E-60	0.318347	0.441	0.091	2.73E-57	EN	FRMD4B
80	2.36E-58	0.707397	0.588	0.199	4.72E-55	EN	SRM
81	2.71E-45	0.395402	0.38	0.104	5.42E-42	EN	FOXO3
82	7.79E-43	0.54251	0.396	0.096	1.56E-39	EN	SERPINE2
83	3.03E-37	0.324371	0.435	0.104	6.07E-34	EN	TTLL7
84	4.47E-25	0.326771	0.572	0.169	8.95E-22	EN	RHOB
85	4.47E-23	0.430262	0.573	0.133	8.94E-20	EN	PPP2R2B
86	3.75E-19	0.439356	0.344	0.091	7.50E-16	EN	MCUR1
87	8.63E-12	0.380793	0.364	0.119	1.73E-08	EN	DLGAP4
88	3.86E-07	0.498475	0.451	0.17	0.000772	EN	MSMO1
89	0.000886	0.317082	0.531	0.145	1	EN	ZNF462
90	0.001322	0.379811	0.491	0.146	1	EN	RNF24
91	0	1.176093	0.843	0.301	0	IN	MAP1B
92	0	0.863669	0.267	0.152	0	IN	VCAN
93	0	0.685366	0.306	0.165	0	IN	RNF24
94	0	0.606377	0.305	0.211	0	IN	DDX6
95	0	0.587995	0.275	0.211	0	IN	MDK
96	1.05E-283	0.607314	0.263	0.177	2.09E-280	IN	FBXO21
97	6.00E-273	0.616947	0.276	0.21	1.20E-269	IN	RHOB
98	3.29E-271	0.726179	0.677	0.288	6.57E-268	IN	TCF4
99	2.30E-196	0.558874	0.378	0.231	4.60E-193	IN	BCL11A
100	1.02E-175	0.641551	0.407	0.299	2.05E-172	IN	ENC1
101	4.35E-175	1.296428	0.326	0.062	8.70E-172	IN	PDE4DIP
102	1.73E-170	0.602025	0.372	0.234	3.45E-167	IN	GPM6B
103	3.09E-168	0.604573	0.757	0.424	6.18E-165	IN	FOS
104	4.13E-161	0.654075	0.394	0.122	8.26E-158	IN	RBP1
105	1.42E-146	0.963071	0.339	0.138	2.85E-143	IN	TMEM123
106	2.34E-122	0.874289	0.381	0.217	4.68E-119	IN	CITED2
107	1.37E-91	0.743314	0.384	0.219	2.73E-88	IN	KLC1
108	9.12E-75	1.675168	0.389	0.023	1.82E-71	IN	PLS3
109	1.00E-74	0.84027	0.41	0.277	2.01E-71	IN	IGFBP2
110	2.07E-71	0.77259	0.999	0.754	4.14E-68	IN	CXCR4
111	4.90E-64	0.711377	0.425	0.238	9.79E-61	IN	APP
112	5.25E-64	0.807829	0.424	0.239	1.05E-60	IN	APLP1
113	3.96E-49	1.093898	0.541	0.209	7.92E-46	IN	PFN2
114	1.36E-27	0.584614	0.457	0.242	2.73E-24	IN	FSCN1
115	5.58E-24	1.118658	0.472	0.111	1.12E-20	IN	MEG3
116	5.81E-15	0.781082	0.508	0.294	1.16E-11	IN	EGR1
117	9.38E-09	0.934276	0.553	0.276	1.88E-05	IN	POLR2J3
118	7.60E-07	0.599253	0.493	0.252	0.001521	IN	FEZ1
119	0.00353	1.132764	0.461	0.211	1	IN	HBA2
120	0.004891	0.843926	0.537	0.226	1	IN	MIAT
121	0	1.014604	1	0.962	0	RG	RPS26
122	0	0.886849	0.789	0.228	0	RG	FDFT1
123	0	0.825342	0.802	0.174	0	RG	TSPAN13
124	0	0.781591	0.734	0.203	0	RG	GADD45G
125	0	0.605244	0.802	0.254	0	RG	IGFBP2
126	0	0.589859	0.869	0.345	0	RG	IER2
127	0	0.567736	0.793	0.277	0	RG	C1orf61
128	0	0.567529	0.814	0.266	0	RG	NFIB
129	0	0.566018	0.922	0.444	0	RG	FOS
130	1.25E-285	0.805904	0.773	0.291	2.50E-282	RG	EGR1
131	5.47E-262	0.74324	0.728	0.208	1.09E-258	RG	STMN4
132	3.04E-251	0.590931	0.307	0.061	6.08E-248	RG	MYC
133	2.03E-230	0.774518	0.721	0.214	4.06E-227	RG	BCL11A
134	1.05E-186	1.101851	0.659	0.163	2.11E-183	RG	MSMO1
135	7.67E-174	0.837303	0.669	0.151	1.53E-170	RG	NELL2
136	9.27E-170	0.833079	0.656	0.186	1.85E-166	RG	SQLE
137	3.38E-145	0.667656	0.713	0.249	6.77E-142	RG	EZR
138	5.61E-110	0.662248	0.292	0.047	1.12E-106	RG	EPB41L3
139	5.28E-105	0.647077	0.332	0.081	1.06E-101	RG	FGFBP3
140	1.25E-89	0.567749	0.382	0.055	2.50E-86	RG	PRDM8
141	3.72E-80	0.587963	0.386	0.088	7.45E-77	RG	EPHB6
142	7.85E-62	0.57208	0.604	0.163	1.57E-58	RG	NFIA
143	3.31E-60	0.589274	0.612	0.15	6.63E-57	RG	PPP2R2B
144	5.69E-56	0.745958	0.334	0.058	1.14E-52	RG	CDK2AP1
145	2.73E-42	0.875257	0.506	0.137	5.47E-39	RG	SSBP2
146	3.01E-31	0.84995	0.505	0.122	6.02E-28	RG	PLK2
147	2.38E-22	0.634789	0.555	0.151	4.75E-19	RG	ACAT2
148	1.99E-18	0.753573	0.556	0.141	3.98E-15	RG	HMGCS1
149	2.60E-09	0.640016	0.437	0.075	5.21E-06	RG	LIMCH1
150	2.78E-09	0.67092	0.52	0.11	5.55E-06	RG	SEZ6
151	0	3.222196	0.767	0.601	0	Macrophage	LYZ
152	0	1.918849	0.971	0.86	0	Macrophage	FTL
153	0	1.883297	0.951	0.858	0	Macrophage	FTH1
154	0	1.563697	0.495	0.285	0	Macrophage	CST3
155	0	1.501245	0.769	0.766	0	Macrophage	HLA-DRB1
156	0	1.290652	0.899	0.857	0	Macrophage	HLA-DRA
157	8.32E-238	1.636765	0.459	0.322	1.66E-234	Macrophage	PSAP
158	1.52E-132	1.097258	0.171	0.504	3.03E-129	Macrophage	GPNMB
159	2.80E-96	1.39112	0.611	0.679	5.60E-93	Macrophage	SAT1
160	5.33E-65	1.083637	0.165	0.4	1.07E-61	Macrophage	CYBB
161	1.47E-60	2.062164	0.475	0.526	2.94E-57	Macrophage	NPC2
162	1.39E-57	1.241721	0.308	0.349	2.79E-54	Macrophage	CTSD
163	1.76E-36	1.577939	0.275	0.557	3.53E-33	Macrophage	PTGDS
164	4.99E-29	1.659169	0.268	0.525	9.99E-26	Macrophage	FCER1G
165	5.06E-27	1.090363	0.479	0.584	1.01E-23	Macrophage	NFKBIA
166	1.27E-25	1.040474	0.185	0.44	2.54E-22	Macrophage	FCGRT
167	2.38E-24	2.544981	0.391	0.47	4.77E-21	Macrophage	C1QB
168	6.06E-24	1.728685	0.249	0.486	1.21E-20	Macrophage	C1QC
169	4.75E-21	1.259864	0.3	0.553	9.50E-18	Macrophage	TYMP
170	6.84E-21	2.155182	0.432	0.549	1.37E-17	Macrophage	TYROBP
171	8.42E-20	1.346076	0.306	0.554	1.68E-16	Macrophage	CTSS
172	8.23E-19	2.600418	0.413	0.53	1.65E-15	Macrophage	C1QA
173	1.49E-18	1.069653	0.203	0.409	2.98E-15	Macrophage	TMEM176B
174	8.04E-14	2.596887	0.437	0.571	1.61E-10	Macrophage	APOE
175	7.43E-06	1.203199	0.177	0.373	0.014856	Macrophage	IER3
176	1.37E-05	1.293348	0.307	0.516	0.027378	Macrophage	CTSB
177	0.000228	2.224462	0.347	0.557	0.455699	Macrophage	APOC1
178	0.000443	1.193603	0.465	0.649	0.8867	Macrophage	NEAT1
179	0.00227	1.939978	0.314	0.515	1	Macrophage	S100A9
180	0.007066	1.649995	0.395	0.567	1	Macrophage	AIF1
181	2.14E-280	1.224818	0.982	0.706	4.29E-277	INP	TUBA1B
182	8.01E-272	0.537767	0.857	0.416	1.60E-268	INP	PLK1
183	1.07E-248	1.254634	0.989	0.755	2.15E-245	INP	HMGB2
184	1.21E-200	1.068064	0.832	0.472	2.42E-197	INP	UBE2C
185	6.38E-181	0.620844	0.812	0.501	1.28E-177	INP	NUSAP1
186	1.21E-165	0.661536	0.845	0.415	2.42E-162	INP	SOX4
187	1.93E-164	0.64619	0.98	0.55	3.85E-161	INP	HMGN2
188	1.20E-109	0.72275	0.722	0.379	2.40E-106	INP	MAD2L1
189	1.15E-97	0.906119	0.705	0.236	2.30E-94	INP	GADD45G
190	2.94E-69	0.743315	0.614	0.072	5.88E-66	INP	ZWINT
191	3.94E-63	0.569061	0.614	0.144	7.88E-60	INP	UBE2S
192	9.37E-61	1.365333	0.707	0.37	1.87E-57	INP	HIST1H4C
193	1.19E-55	0.605958	0.639	0.089	2.37E-52	INP	RRM1
194	1.07E-50	1.254713	0.64	0.285	2.14E-47	INP	TOP2A
195	4.06E-40	0.640039	0.612	0.231	8.12E-37	INP	H1F0
196	2.21E-32	0.551687	0.639	0.443	4.42E-29	INP	TYMS
197	1.10E-31	0.706108	0.66	0.234	2.19E-28	INP	TUBB4B
198	1.36E-28	0.550829	0.403	0.295	2.72E-25	INP	MCM2
199	5.40E-23	0.568466	0.44	0.348	1.08E-19	INP	CDCA8
200	2.65E-21	0.666428	0.614	0.479	5.30E-18	INP	CDK1
201	6.59E-21	0.568269	0.608	0.375	1.32E-17	INP	MKI67
202	7.71E-21	0.686143	0.563	0.138	1.54E-17	INP	NDC80
203	3.70E-15	0.870081	0.6	0.482	7.39E-12	INP	CENPF
204	2.45E-14	0.594674	0.44	0.143	4.90E-11	INP	KIF23
205	2.05E-11	0.789692	0.544	0.212	4.11E-08	INP	GTSE1
206	2.02E-10	0.754588	0.75	0.475	4.05E-07	INP	CENPA
207	5.51E-10	0.732157	0.549	0.227	1.10E-06	INP	PRC1
208	2.96E-09	0.591412	0.614	0.445	5.91E-06	INP	AURKB
209	4.96E-09	0.805126	0.548	0.223	9.92E-06	INP	CCNB2
210	1.61E-07	0.59184	0.366	0.105	0.000322	INP	CKAP2L
211	0	3.70269	0.822	0.285	0	Dendritic_cell	CST3
212	0	1.973041	0.961	0.857	0	Dendritic_cell	HLA-DRA
213	0	1.753925	0.994	0.944	0	Dendritic_cell	CD74
214	2.37E-299	2.098112	0.882	0.752	4.73E-296	Dendritic_cell	HLA-DPB1
215	5.01E-277	2.015071	0.878	0.763	1.00E-273	Dendritic_cell	HLA-DRB1
216	4.61E-252	0.763202	1	1	9.22E-249	Dendritic_cell	TMSB4X
217	5.28E-246	2.048847	0.828	0.712	1.06E-242	Dendritic_cell	HLA-DPA1
218	7.11E-106	1.717829	0.412	0.286	1.42E-102	Dendritic_cell	SNX3
219	9.82E-98	2.294816	0.65	0.61	1.96E-94	Dendritic_cell	LYZ
220	2.67E-85	0.915472	0.102	0.491	5.34E-82	Dendritic_cell	FGL2
221	4.65E-76	1.082696	0.539	0.452	9.30E-73	Dendritic_cell	VIM
222	8.29E-76	0.979316	0.128	0.513	1.66E-72	Dendritic_cell	FGD2
223	1.08E-70	0.838986	0.155	0.547	2.16E-67	Dendritic_cell	HLA-DMB
224	7.55E-64	1.027126	0.124	0.471	1.51E-60	Dendritic_cell	SPI1
225	2.23E-58	0.887947	0.125	0.492	4.47E-55	Dendritic_cell	PTPRE
226	9.41E-58	0.909367	0.173	0.546	1.88E-54	Dendritic_cell	TYMP
227	3.79E-40	0.762269	0.221	0.572	7.57E-37	Dendritic_cell	ANXA2
228	2.84E-32	1.462134	0.187	0.477	5.67E-29	Dendritic_cell	C1orf162
229	3.35E-25	0.777521	0.267	0.333	6.70E-22	Dendritic_cell	PSAP
230	7.26E-25	1.504547	0.206	0.502	1.45E-21	Dendritic_cell	IRF8
231	1.97E-23	0.969484	0.275	0.594	3.94E-20	Dendritic_cell	RGS10
232	1.22E-18	0.90593	0.269	0.572	2.44E-15	Dendritic_cell	TAGLN2
233	3.75E-18	0.831439	0.259	0.562	7.51E-15	Dendritic_cell	CKLF
234	4.01E-14	0.852343	0.332	0.639	8.02E-11	Dendritic_cell	S100A11
235	1.52E-08	0.993495	0.345	0.623	3.03E-05	Dendritic_cell	HLA-DMA
236	1.71E-08	1.575868	0.277	0.526	3.43E-05	Dendritic_cell	RGCC
237	4.19E-07	2.373785	0.364	0.46	0.000838	Dendritic_cell	CPVL
238	2.61E-05	1.645703	0.447	0.606	0.052238	Dendritic_cell	S100A10
239	0.002237	1.138636	0.473	0.656	1	Dendritic_cell	LGALS1
240	0.008281	1.435878	0.339	0.562	1	Dendritic_cell	AIF1
241	6.49E-303	1.904416	0.868	0.068	1.30E-299	ENP	EOMES
242	3.29E-225	0.42582	0.979	0.415	6.58E-222	ENP	SOX4
243	3.75E-154	0.400463	0.254	0.196	7.51E-151	ENP	RASGRP1
244	4.14E-138	0.625844	0.983	0.517	8.28E-135	ENP	PRDX1
245	1.66E-118	0.440764	0.989	0.553	3.32E-115	ENP	STK17A
246	2.24E-109	0.505076	1	1	4.48E-106	ENP	TMSB4X
247	1.66E-106	0.539125	0.73	0.19	3.33E-103	ENP	NFIA
248	3.75E-105	0.985227	0.743	0.13	7.51E-102	ENP	CORO1C
249	4.56E-98	0.668252	0.798	0.29	9.11E-95	ENP	IGFBP2
250	1.04E-80	0.795455	0.733	0.267	2.08E-77	ENP	FDFT1
251	2.70E-68	0.466922	0.655	0.261	5.41E-65	ENP	PFDN4
252	6.93E-68	0.500178	0.329	0.077	1.39E-64	ENP	CDK2AP1
253	1.14E-65	0.664417	0.787	0.323	2.28E-62	ENP	EGR1
254	3.54E-57	0.400239	0.251	0.079	7.07E-54	ENP	MYC
255	7.72E-56	0.647243	0.278	0.033	1.54E-52	ENP	LYPD1
256	1.33E-48	0.599518	0.999	0.964	2.65E-45	ENP	RPS26
257	3.05E-45	0.681836	0.69	0.158	6.10E-42	ENP	PHLDA1
258	1.19E-44	0.424229	0.358	0.054	2.37E-41	ENP	ZEB1
259	2.62E-41	0.652534	0.688	0.196	5.24E-38	ENP	CCND2
260	3.48E-34	0.462049	0.645	0.232	6.95E-31	ENP	H1F0
261	1.04E-32	0.885923	0.614	0.091	2.08E-29	ENP	ADGRG1
262	2.88E-25	0.530315	0.631	0.145	5.75E-22	ENP	IVNS1ABP
263	1.94E-24	0.511057	0.637	0.178	3.89E-21	ENP	FBLN1
264	2.41E-19	0.469211	0.428	0.113	4.83E-16	ENP	OLFM2
265	9.40E-14	0.477702	0.624	0.239	1.88E-10	ENP	CITED2
266	3.62E-11	0.476086	0.453	0.167	7.23E-08	ENP	VCAN
267	2.04E-08	0.734849	0.542	0.164	4.07E-05	ENP	RBP1
268	3.21E-06	0.507731	0.578	0.136	0.006419	ENP	SEZ6
269	0.000191	0.506387	0.404	0.191	0.382023	ENP	ZNF462
270	0.00236	0.397547	0.407	0.225	1	ENP	TSPAN13
271	0	1.614523	0.928	0.204	0	Astrocyte	PTN
272	0	0.810443	0.285	0.008	0	Astrocyte	TFPI
273	1.42E-236	0.755542	0.333	0.025	2.84E-233	Astrocyte	EEPD1
274	5.24E-167	1.068249	0.78	0.059	1.05E-163	Astrocyte	TTYH1
275	7.75E-141	0.870803	0.962	0.444	1.55E-137	Astrocyte	VIM
276	1.32E-103	1.291745	0.723	0.053	2.64E-100	Astrocyte	PON2
277	2.42E-94	1.11626	0.709	0.062	4.85E-91	Astrocyte	CLU
278	7.41E-93	0.643001	0.308	0.115	1.48E-89	Astrocyte	PDGFD
279	2.38E-82	1.148098	0.704	0.12	4.76E-79	Astrocyte	PSAT1
280	7.00E-73	0.700365	0.735	0.225	1.40E-69	Astrocyte	CNN3
281	2.07E-69	0.718801	0.715	0.127	4.15E-66	Astrocyte	PEA15
282	5.00E-56	0.884916	0.702	0.167	1.00E-52	Astrocyte	HMGCS1
283	6.36E-49	0.936089	0.648	0.055	1.27E-45	Astrocyte	SLC1A3
284	3.06E-47	0.800403	0.661	0.125	6.12E-44	Astrocyte	ZFP36L1
285	4.65E-45	1.404385	0.739	0.517	9.31E-42	Astrocyte	HOPX
286	2.66E-44	1.02501	0.663	0.092	5.32E-41	Astrocyte	FGFBP3
287	6.01E-40	0.915995	0.637	0.166	1.20E-36	Astrocyte	GATM
288	1.20E-39	0.647976	0.909	0.67	2.41E-36	Astrocyte	SAT1
289	2.72E-34	0.688902	0.956	0.673	5.45E-31	Astrocyte	JUNB
290	3.10E-21	0.672502	0.603	0.178	6.21E-18	Astrocyte	ACAT2
291	4.41E-19	0.760404	0.597	0.048	8.83E-16	Astrocyte	PHGDH
292	1.51E-18	0.98821	0.586	0.017	3.01E-15	Astrocyte	PLPP3
293	2.75E-10	0.685859	0.424	0.157	5.50E-07	Astrocyte	ABHD3
294	7.99E-10	1.086946	0.56	0.025	1.60E-06	Astrocyte	MOXD1
295	3.06E-09	0.693082	0.427	0.072	6.12E-06	Astrocyte	CDCA7
296	4.94E-06	0.698006	0.398	0.047	0.009875	Astrocyte	B3GAT2
297	0.001865	1.098759	0.458	0.015	1	Astrocyte	GFAP
298	0.002115	0.874327	0.512	0.065	1	Astrocyte	ITM2C
299	0.002679	0.820295	0.472	0.063	1	Astrocyte	MFGE8
300	0.009071	0.635672	0.532	0.051	1	Astrocyte	SPARC
301	3.79E-185	3.805686	0.667	0.153	7.59E-182	Oligodendrocyte	PLP1
302	2.58E-139	2.539978	0.388	0.052	5.16E-136	Oligodendrocyte	PPP1R14A
303	2.21E-136	2.272036	0.305	0.05	4.41E-133	Oligodendrocyte	CLDN11
304	8.42E-96	2.68664	0.4	0.151	1.68E-92	Oligodendrocyte	CRYAB
305	1.08E-95	1.927866	0.326	0.108	2.17E-92	Oligodendrocyte	CNP
306	1.04E-52	0.824873	0.877	0.864	2.09E-49	Oligodendrocyte	FTH1
307	4.26E-43	0.577026	0.119	0.524	8.52E-40	Oligodendrocyte	RGCC
308	7.87E-34	0.612683	0.087	0.463	1.57E-30	Oligodendrocyte	AFMID
309	1.78E-32	0.990412	0.354	0.309	3.55E-29	Oligodendrocyte	CD63
310	1.92E-30	2.721724	0.445	0.373	3.83E-27	Oligodendrocyte	MBP
311	6.38E-30	0.811037	0.159	0.525	1.28E-26	Oligodendrocyte	APOL2
312	1.56E-29	0.646876	0.174	0.546	3.12E-26	Oligodendrocyte	CD82
313	5.02E-25	0.980243	0.184	0.539	1.00E-21	Oligodendrocyte	ISG15
314	1.30E-22	0.928424	0.191	0.521	2.60E-19	Oligodendrocyte	IFI6
315	2.36E-21	1.216557	0.157	0.478	4.72E-18	Oligodendrocyte	APOD
316	5.57E-21	1.469877	0.25	0.26	1.11E-17	Oligodendrocyte	GPM6B
317	2.73E-18	2.478841	0.504	0.54	5.45E-15	Oligodendrocyte	PTGDS
318	3.43E-18	0.579492	0.066	0.369	6.86E-15	Oligodendrocyte	UGT8
319	3.00E-17	0.631393	0.261	0.606	6.00E-14	Oligodendrocyte	LCP1
320	8.04E-14	0.671266	0.174	0.254	1.61E-10	Oligodendrocyte	HSPA1A
321	2.68E-12	0.717355	0.305	0.607	5.35E-09	Oligodendrocyte	IFI16
322	1.20E-11	1.115476	0.131	0.413	2.41E-08	Oligodendrocyte	CA2
323	1.81E-11	0.574622	0.309	0.637	3.63E-08	Oligodendrocyte	DUSP2
324	1.90E-08	1.397535	0.155	0.347	3.80E-05	Oligodendrocyte	TF
325	2.35E-06	0.913646	0.345	0.641	0.004702	Oligodendrocyte	NEAT1
326	3.63E-05	0.628424	0.333	0.603	0.072666	Oligodendrocyte	CTNNB1
327	0.000149	0.609116	0.379	0.663	0.297713	Oligodendrocyte	LTB
328	0.000493	0.578326	0.347	0.475	0.986082	Oligodendrocyte	DBI
329	0.005235	1.259594	0.237	0.463	1	Oligodendrocyte	TUBA1A
330	0.005577	0.613952	0.125	0.275	1	Oligodendrocyte	APLP1
331	0	2.835661	0.771	0.117	0	Miningeal_cell	DCN
332	0	2.504664	0.719	0.03	0	Miningeal_cell	LUM
333	0	1.904108	0.657	0.012	0	Miningeal_cell	C1S
334	0	1.493626	0.592	0.012	0	Miningeal_cell	CCL2
335	1.58E-220	4.125275	0.881	0.227	3.17E-217	Miningeal_cell	IGFBP7
336	2.97E-190	1.368758	0.61	0.069	5.93E-187	Miningeal_cell	IFI27
337	3.51E-171	1.947011	0.668	0.039	7.01E-168	Miningeal_cell	MGP
338	7.74E-170	1.06796	0.561	0.009	1.55E-166	Miningeal_cell	NNMT
339	7.13E-167	1.004315	0.592	0.022	1.43E-163	Miningeal_cell	PCOLCE
340	2.65E-156	3.395456	0.826	0.471	5.31E-153	Miningeal_cell	APOD
341	2.14E-155	4.564732	0.865	0.471	4.28E-152	Miningeal_cell	TIMP1
342	5.67E-155	1.64002	0.636	0.055	1.13E-151	Miningeal_cell	FN1
343	1.80E-140	1.158199	0.569	0.017	3.59E-137	Miningeal_cell	COL1A2
344	1.28E-103	1.593108	0.577	0.11	2.56E-100	Miningeal_cell	C1R
345	6.72E-99	1.251208	0.629	0.216	1.34E-95	Miningeal_cell	MT1X
346	4.92E-92	2.378998	0.727	0.294	9.83E-89	Miningeal_cell	CST3
347	4.77E-79	2.261652	0.73	0.538	9.55E-76	Miningeal_cell	PTGDS
348	3.54E-73	1.358363	0.395	0.009	7.07E-70	Miningeal_cell	TFPI
349	1.67E-72	2.351417	0.668	0.407	3.34E-69	Miningeal_cell	IFITM3
350	1.81E-63	1.345363	0.701	0.306	3.62E-60	Miningeal_cell	CD63
351	8.62E-61	1.044653	0.423	0.062	1.72E-57	Miningeal_cell	PON2
352	1.81E-57	1.256814	0.423	0.071	3.62E-54	Miningeal_cell	CLU
353	1.73E-49	1.793979	0.621	0.299	3.46E-46	Miningeal_cell	IGFBP2
354	3.77E-38	1.042001	0.468	0.186	7.55E-35	Miningeal_cell	FBLN1
355	6.80E-29	1.368242	0.834	0.717	1.36E-25	Miningeal_cell	MT2A
356	9.04E-23	0.933653	0.371	0.161	1.81E-19	Miningeal_cell	LGALS3BP
357	6.99E-19	1.766221	0.361	0.018	1.40E-15	Miningeal_cell	TIMP3
358	2.21E-14	0.944842	0.592	0.522	4.42E-11	Miningeal_cell	NPC2
359	6.44E-14	1.087425	0.332	0.13	1.29E-10	Miningeal_cell	CALD1
360	3.21E-12	0.999224	0.242	0.363	6.42E-09	Miningeal_cell	ID3
361	1.06E-196	1.026474	0.692	0.019	2.12E-193	OPC	ALCAM
362	8.54E-161	0.926713	0.842	0.053	1.71E-157	OPC	PPP1R14A
363	3.67E-97	2.660542	0.952	0.011	7.34E-94	OPC	OLIG1
364	3.59E-76	3.156376	0.945	0.473	7.19E-73	OPC	APOD
365	1.40E-46	0.717711	0.993	0.489	2.79E-43	OPC	DBNDD2
366	1.88E-41	1.5439	0.808	0.04	3.76E-38	OPC	SCRG1
367	2.35E-22	1.003988	0.767	0.139	4.69E-19	OPC	SCD5
368	2.78E-22	2.741979	0.699	0.032	5.57E-19	OPC	S100B
369	9.43E-20	0.752623	0.788	0.321	1.89E-16	OPC	C1orf61
370	3.17E-17	1.728659	0.664	0.071	6.34E-14	OPC	ITM2C
371	1.86E-15	0.728462	0.603	0.139	3.73E-12	OPC	ATP1B1
372	2.31E-15	1.214095	0.603	0.005	4.61E-12	OPC	CMTM5
373	4.46E-15	0.722829	0.712	0.216	8.92E-12	OPC	PTN
374	1.62E-11	0.873344	0.329	0.044	3.23E-08	OPC	DUSP6
375	2.19E-11	0.962117	0.719	0.3	4.38E-08	OPC	IGFBP2
376	4.85E-11	1.254304	0.623	0.101	9.69E-08	OPC	SIRT2
377	5.32E-11	1.529403	0.637	0.177	1.06E-07	OPC	MEG3
378	7.61E-10	1.305709	0.363	0.152	1.52E-06	OPC	BAMBI
379	2.01E-09	0.717718	0.527	0.042	4.01E-06	OPC	RAB40B
380	1.04E-08	0.840125	0.363	0.19	2.07E-05	OPC	ETV1
381	2.32E-07	1.364814	0.548	0.012	0.000465	OPC	PLLP
382	2.13E-06	0.891471	0.582	0.252	0.004265	OPC	STMN4
383	2.34E-06	1.240183	0.63	0.134	0.004677	OPC	SERPINE2
384	7.07E-06	1.395596	0.575	0.088	0.014136	OPC	PCDH9
385	0.000158	1.329678	0.575	0.064	0.315588	OPC	PON2
386	0.000168	0.831965	0.61	0.169	0.336561	OPC	PHLDA1
387	0.000635	1.61912	0.582	0.104	1	OPC	DLL3
388	0.001036	0.734948	0.5	0.5	1	OPC	CTHRC1
389	0.001612	0.761199	0.425	0.061	1	OPC	NCALD
390	0.00172	1.082534	0.575	0.148	1	OPC	CLDND1

**Table 2:** The proportion of various subgroups in PCNSL and normal samples.

	cancer	normal	cancer_per	normal_per	Total	%Total
B_cell	8597	133	0.213209	0.003298	8730	0.216507
T_cell	7782	136	0.192996	0.003373	7918	0.196369
EN	7	5279	0.000174	0.130921	5286	0.131095
IN	8	7507	0.000198	0.186176	7515	0.186375
RG	0	3538	0	0.087744	3538	0.087744
Macrophage	2252	262	0.05585	0.006498	2514	0.062348
INP	16	1129	0.000397	0.028	1145	0.028396
Dendritic_cell	998	0	0.024751	0	998	0.024751
ENP	9	897	0.000223	0.022246	906	0.022469
Astrocyte	3	766	7.44E-05	0.018997	769	0.019071
Oligodendrocyte	454	18	0.011259	0.000446	472	0.011706
Miningeal_cell	179	206	0.004439	0.005109	385	0.009548
OPC	2	144	4.96E-05	0.003571	146	0.003621
	20307	20015			40322	

### The Heterogeneity of B Cells in PCNSL and Their Communication Interaction With Other Immune Cells

**Figure 2:**
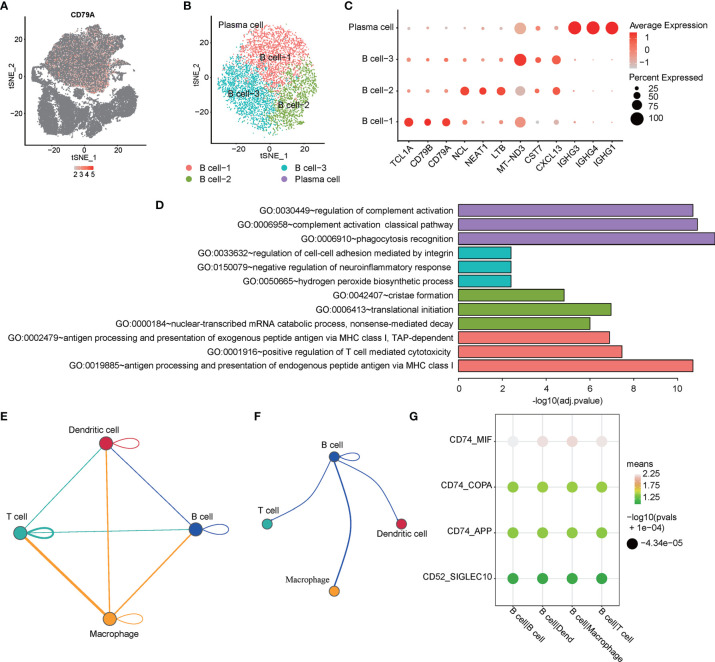
Single-cell analysis of heterogeneity for B cell and cell communication. **(A)** The expression distribution of the B cell maker gen -- CD79A in all cells. **(B)** tSNE visualization describes the re-cluster results of B cell subgroups. **(C)** The bubble chart shows that selected three marker genes are selected for each cell subgroup, and their expression in all cell subpopulations is plotted. **(D)** The top3 representative GO functional annotation categories of marker genes for each sub-population. **(E)** The interactions among immune-related cell subpopulations: B cell, T cell, macrophage, and dendritic cell subpopulation. **(F)** The interaction pairs of B cell with T cell, macrophage, and dendritic cell, respectively. **(G)** The ligand-receptor pairs are contributing to the signaling from B cells to the other three clusters.

Malignant proliferation and abnormal differentiation of B cells (toward plasma cells) are the main pathological characteristics of PCNSL. Molecular targeted therapy targeting B cells has also been considered as a promising way to treat PCNSL in recent years. Nevertheless, the heterogeneity of B cells in PCNSL remains unclear. Therefore, we first analyzed the heterogeneity of B-cell subtypes and genetic characteristics in PCNSL from single-cell resolution. *CD79A* served as a typical marker gene of the B cell population in total PCNSL cells (**Figure 2A**). Then, the identical methods with cell population detection were used to re-cluster B cells into four distinct subclusters: B cell-1, B cell-2, B cell-3, and plasma cell (**Figure 2B**). We identified the marker genes for each subcluster among the B cell population (**Figure 2C** and **Supplementary Table 1**). The functional annotations of each subcluster were counted (**Supplementary Table 2**) and the three significant pathways are shown in **Figure 2D**. The B cell-1 cluster highly expressed marker genes (e.g., *CD79A*, *CD79B*, *TCL1A*) of the classical B cells. The B cell-2 cluster was significantly marked by, e.g., NCL, LTB, NEAT1, and enriched functional pathways of antigen processing and presentation of endogenous peptide antigen *via* MHC class I and positive regulation of T cell-mediated cytotoxicity, suggesting that these B cells have strong antigen–antibody immunity and synergistic disturbance with T cells. The B cell-3 cluster (e.g., CST7, CXCL13, MT-ND3) indicated functions of cell–cell adhesion regulation, neuroinflammation response, and hydrogen peroxide biosynthetic process. Plasma cells (e.g., IGHG1, IGHG3, IGHG4) played a significant role in complement activation.

Cell–cell communication is a fundamental process that shapes biological tissue. In particular, ligand–receptor pairs can infer intercellular communication from the coordinated expression of their homologous genes (32). Here, we focused on four classes of immune cell subpopulations and immune-related genes. **Figure 2E** shows the interactions among immune-related cell subpopulations: B cell, T cell, macrophage, and dendritic cell subpopulation. The circle edge width is proportional to the number of cells in each cell cluster and the communication score between interacting cell clusters, respectively. The circle plot (**Figure 2F**) showed the interactions of B cell with T cell, macrophage, and dendritic cell, respectively. The bubble plots (**Figure 2G**) show the ligand–receptor pairs contributing to the signaling from B cells to other three clusters. The circle plots show that the interaction between macrophage and T cell has the most count numbers among others. Interestingly, we identified that significant target CD74 with MIF, COPA, and APP might participate the process of B cell interacting with T cell, macrophage, and dendritic cell based on the CellPhoneDB results, and CD74–MIF interaction was the most significant among these.

### The Heterogeneity of T Cells and Dendritic Cells in PCNSL

**Figure 3:**
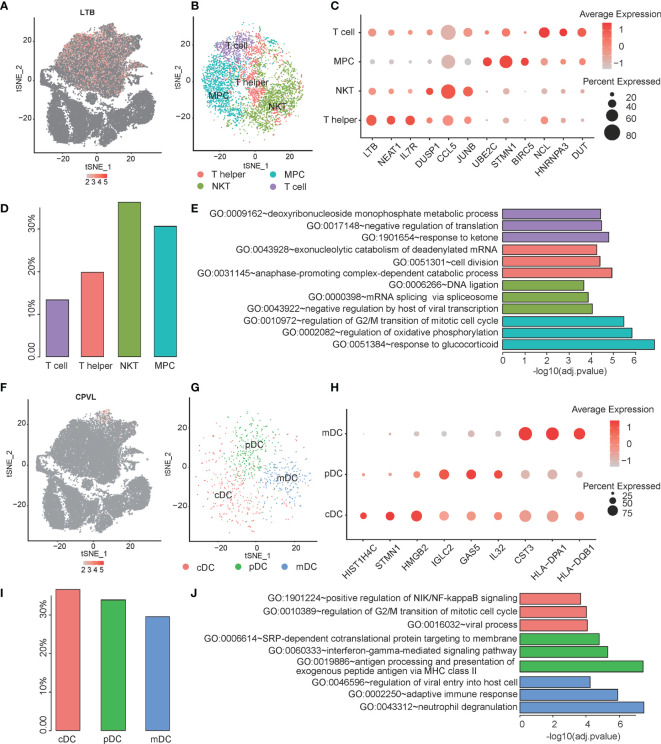
Single-cell analysis of heterogeneity for T cell and Dendritic cell sub-population. **(A)** The expression distribution of the T cell maker gene -- LTB in all cells. **(B)** The tSNE visualization describes the re-cluster results of T cell subgroups. **(C)** The bubble chart shows that selected three marker genes are selected for each cell subgroup, and their expression in all cell subpopulations is plotted. **(D)** The proportion of each subpopulation for T cell. **(E)** The top three significant GO categories of marker genes for each subgroup are enriched. **(F)** The expression distribution of the Dendritic cell maker gene -- CPVL in all cells. **(G)** The tSNE visualization describes the re-cluster results of dendritic cell subgroups. **(H)** The bubble chart shows that selected three marker genes are selected for each cell subgroup, and their expression in all cell subpopulations is plotted. **(I)** The proportion of each subpopulation for dendritic cells. **(J)** The top three significant GO categories of marker genes for each subgroup are enriched.

T cells and dendritic cells have a high proportion in PCNSL and are closely related to the immune microenvironment of PCNSL. The subpopulations of T cells and dendritic cells in PCNSL were analyzed. *LTB* serves as a marker gene of the T cell population (**Figure 3A**). Then, T cells were re-clustered into four distinct subclusters: naive T cells, natural killer T (NKT) cells, T helper cells, and MPCs (**Figure 3B**). Further clustering of the T cell subpopulation in PCNSL gave rise to four subpopulations with specific gene signatures, including T helper cell group (LTB, NEAT1, and IL7R), NKT cell group (DUSP1, CCL5, and JUNB), MPC cell group (UBE2C, STMN1, and BIRC5), and classical T cell group (NCL, HNRNPA3, and DUT). Bubble heatmap showing expression levels of selected signature genes in ESCA. Dot size indicates a fraction of expressing cells, colored based on normalized expression levels (**Figure 3C**). The proportions of these subtypes are shown in **Figure 3D**. The marker genes were identified for each subcluster among the T cell population (**Supplementary Table 3**), and the three typical marker genes for annotation are shown in a bubble plot (**Figure 3D**). Three significant function pathways enriched by the marker genes of the four subclusters are shown in **Figure 3E** (**Supplementary Table 4**). DEGs of MPC were highly enriched in the G2/M transition of the mitotic cell cycle pathway, which was closely related to the proliferation and differentiation of various lymphocytes in PCNSL and might be the main source of tumor germination.

The dendritic cell population was labeled by a marker gene *CPVL* (**Figure 3F**). Then, the dendritic cells were re-clustered into three distinct clusters: conventional dendritic cell (cDC), myeloid dendritic cell (mDC), and plasmacytoid dendritic cell (pDC) (**Figure 3G**). We identified the marker genes for each subcluster among the dendritic cell population (**Supplementary Table 5**). The typical marker genes for annotation are shown in a bubble plot (cDC: e.g., STMN1, HMGB2, HIST1H4C; mDC: e.g., CST3, HLA-DPA1, HLA-DQB1; pDC: IGLC2, GAS5, IL32) (**Figure 3H** and **Supplementary Table 5**). The proportions of the subclusters are shown in **Figure 3I**. Evaluating known pathway expression in every three subpopulations of dendritic cell group using GO enrichment analysis revealed strong enrichment of regulation of viral entry into host cell, adaptive immune response, and neutrophil degranulation for mDC subpopulation; SRP-dependent cotranslational protein targeting to membrane , interferon-gamma-mediated signaling pathway, and antigen processing and presentation of exogenous peptide antigen via MHC class II for pDC; and positive regulation of NIK/NF-kappaB signaling, regulation of G2/M transition of mitotic cell cycle, and viral process in cDC. The function pathways of the marker genes enriched by GO analysis are shown in **Figure 3J** and **Supplementary Table 6**.

### Identification and Variation Analysis of Macrophage Between PCNSL and Normal Brain

**Figure 4:**
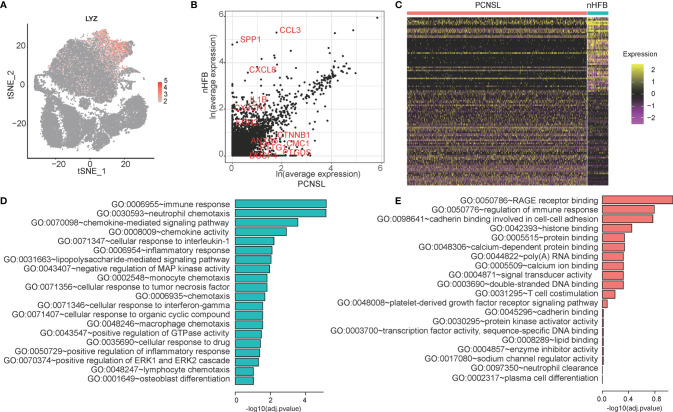
The differential expression analysis of macrophage between cancer and normal samples. **(A)** The expression distribution of the macrophage cell marker gene -- LYZ in all cells. **(B)** The scatter plot of genes selected five significant DEGs in the macrophage cell population between cancer and normal samples. **(C)** The heatmap of DEGs of macrophage. **(D, E)** The GO categories of down-regulated genes (green) and up-regulated genes (red) in PCNSL samples compared with normal samples.

It is noted that macrophage in PCNSL and HFB shows obvious difference in cell proportion and distribution. Then they may also have some differences in function. LYZ was used as a marker gene of the macrophage population (**Figure 4A**). NFKBIA (log2FC = -0.38; adj. p-value = 1.32E-8), BTG2 (log2FC = -0.59, adj. p-value = 1.44E-18), NFKBIZ (log2FC = -0.28, adj. p-value = 1.18E-20), TLR4 (log2FC = –0.53, adj. p-value = 7.35E-34), and TLR10 (log2FC = -0.54, adj. p-value = 3.69E-40) are selected for visualization of the differentially expressed between PCNSL and nHFB samples (**Figure 4B**). There are 143 DEGs between cancer and normal cells. Fifty genes are upregulated in cancer cells compared to the normal cells, while 93 genes are downregulated in cancer cells compared to normal cells (**Figure 4C** and **Table 3**). Moreover, the top 20 enriched functional pathways of these DEGs are shown in **Figures 4D, E**. Compared with PCNSL, macrophages in nHFB exhibit normal immune function, and the un-regulated DEGs are mainly enriched in pathways including immune response, neutrophil chemotaxis, and chemokine-mediated signaling pathway (**Figure 4D**). However, this classic immune function of macrophage in PCNSL was significantly downregulated, and no significant pathways were found (**Figure 4E**). These results suggest that the immune function of macrophages in PCNSL is significantly suppressed.

**Table 3:** The DEGs between cancer and normal samples in macrophage cell subpopulation.

	p_val	avg_log2FC	pct.1	pct.2	p_val_adj
KLRC2	0	0.391239	0.037	0.969	0
VCAM1	0	0.366472	0.044	1	0
DBNDD2	0	0.277182	0.032	0.924	0
CRIP2	0	-0.29879	0	0.111	0
FGL2	0	0.487956	0.051	0.969	0
P2RX5	0	0.499376	0.054	1	0
CNOT6L	0	0.410654	0.048	0.992	0
MRPL38	0	0.314333	0.037	1	0
CPM	1.04E-303	0.308921	0.056	0.996	2.08E-300
MT1E	5.78E-300	0.556736	0.057	0.996	1.16E-296
APOL2	6.86E-300	0.444724	0.059	1	1.37E-296
TRDC	4.55E-284	0.563092	0.046	0.889	9.10E-281
IQGAP2	9.89E-281	0.558543	0.055	0.977	1.98E-277
RAB11FIP1	1.22E-278	0.35921	0.032	0.107	2.44E-275
SDC4	3.20E-277	0.389471	0.038	0.874	6.40E-274
DZIP3	1.85E-275	0.311801	0.027	0.115	3.70E-272
FAR2	7.90E-275	0.264586	0.009	0.134	1.58E-271
CXCL8	6.84E-17	-1.66317	0.071	0.63	1.37E-13
MGST1	1.69E-269	0.457422	0.063	0.985	3.38E-266
TRIAP1	1.75E-265	0.279175	0.041	0.107	3.51E-262
CDCA7L	1.96E-264	0.539232	0.056	0.966	3.92E-261
CDK6	2.95E-264	0.279297	0.038	0.927	5.91E-261
TRADD	4.66E-259	0.410523	0.047	0.107	9.31E-256
SMC2	8.43E-257	0.404236	0.041	0.115	1.69E-253
BTG3	3.07E-256	0.416585	0.041	0.115	6.14E-253
SRSF8	5.57E-254	0.395906	0.047	0.111	1.11E-250
GMNN	6.60E-253	0.25786	0.032	0.126	1.32E-249
TRIP12	1.55E-250	0.252673	0.027	0.134	3.10E-247
POLD2	1.34E-245	0.281814	0.046	0.118	2.68E-242
TSPAN13	1.39E-244	-0.36467	0.005	0.16	2.78E-241
ISG15	1.23E-242	0.472744	0.082	1	2.45E-239
RHOT1	5.52E-242	-0.28244	0.014	0.153	1.10E-238
STMN4	2.41E-241	-0.42538	0	0.168	4.82E-238
MRTO4	1.43E-240	0.374274	0.039	0.13	2.87E-237
HSPA1A	2.24E-21	-1.54645	0.127	0.691	4.47E-18
UBE2S	2.27E-238	0.25598	0.04	0.13	4.55E-235
HRH2	7.11E-235	-0.26739	0.015	0.844	1.42E-231
PROCR	1.33E-232	-0.26572	0.001	0.172	2.67E-229
FGFBP3	7.58E-232	-0.26347	0.003	0.172	1.52E-228
SH2B2	1.37E-230	-0.25018	0.003	0.821	2.74E-227
SMC4	4.80E-229	0.421929	0.083	0.989	9.60E-226
RGS16	6.35E-227	-0.37795	0.008	0.168	1.27E-223
DNMT1	1.82E-226	0.512429	0.065	0.118	3.64E-223
CD82	2.17E-225	0.508679	0.091	1	4.34E-222
C1QB	7.92E-73	-1.43203	0.323	0.973	1.58E-69
IRF9	1.33E-223	0.388697	0.06	0.939	2.65E-220
IFI6	4.23E-222	0.536596	0.092	1	8.47E-219
CCL3	2.11E-44	-1.37171	0.317	0.939	4.23E-41
CXCL3	5.97E-213	0.415241	0.051	0.84	1.19E-209
C1QC	1.63E-98	-1.36287	0.169	0.935	3.26E-95
AURKB	2.36E-212	0.263125	0.03	0.13	4.72E-209
CCL2	2.28E-77	-1.34748	0.002	0.294	4.57E-74
IL1B	2.10E-48	-1.27349	0.062	0.71	4.20E-45
BLVRA	1.38E-209	0.278868	0.043	0.153	2.77E-206
CORO1C	2.63E-208	-0.30889	0.013	0.179	5.25E-205
MT1X	9.56E-208	0.533701	0.098	0.996	1.91E-204
METTL5	6.09E-199	0.304352	0.05	0.156	1.22E-195
MCM7	1.11E-195	0.331787	0.063	0.149	2.22E-192
RARRES2	3.44E-193	0.518063	0.033	0.168	6.89E-190
TMEM161B-AS1	1.06E-192	-0.33052	0.015	0.191	2.12E-189
FRMD4B	2.23E-192	-0.27404	0.011	0.195	4.45E-189
MRPS35	3.35E-188	0.312066	0.053	0.164	6.70E-185
CCL4	2.38E-14	-1.25075	0.536	0.897	4.75E-11
C1QA	1.24E-65	-1.15028	0.345	1	2.48E-62
PAQR8	3.28E-178	-0.27928	0.008	0.206	6.56E-175
ASCC3	8.82E-178	0.253727	0.02	0.191	1.76E-174
BIRC5	9.26E-174	0.264945	0.047	0.866	1.85E-170
HES6	1.53E-171	-0.3113	0.004	0.218	3.05E-168
ADGRG1	3.75E-171	-0.40328	0.004	0.218	7.49E-168
MDK	3.90E-170	-0.33822	0.001	0.221	7.80E-167
CHD7	1.21E-169	-0.35356	0.016	0.21	2.42E-166
CCL4L2	1.28E-24	-0.98075	0.306	0.878	2.56E-21
LINC00996	2.36E-165	-0.4	0.004	0.221	4.73E-162
SGK1	5.45E-64	-0.97431	0.052	0.725	1.09E-60
RBM42	3.92E-163	0.298274	0.052	0.191	7.84E-160
UBALD2	2.08E-162	0.296831	0.059	0.187	4.17E-159
TIMP1	3.39E-161	0.493413	0.089	0.927	6.77E-158
SLC11A1	7.70E-160	-0.41594	0.004	0.221	1.54E-156
CD74	5.47E-10	-0.96212	0.964	1	1.09E-06
GADD45G	2.17E-159	-0.35941	0.008	0.225	4.35E-156
RGS1	2.82E-14	-0.93902	0.513	0.954	5.64E-11
ITGB3BP	2.78E-155	0.370799	0.04	0.206	5.55E-152
HIST1H2BG	3.18E-89	-0.90098	0.004	0.298	6.36E-86
TRAF5	9.07E-152	0.268677	0.028	0.183	1.81E-148
STK17A	1.08E-148	0.304351	0.14	1	2.16E-145
PRPF31	1.41E-148	0.342162	0.059	0.202	2.82E-145
FN1	3.78E-148	0.292025	0.022	0.225	7.55E-145
CD180	9.33E-147	-0.37612	0.006	0.202	1.87E-143
RNASEH2B	1.55E-146	0.476378	0.076	0.195	3.09E-143
UQCC2	2.24E-146	0.345537	0.063	0.202	4.48E-143
FCGR1A	2.72E-59	-0.88509	0.038	0.71	5.45E-56
VAMP5	4.93E-142	0.563777	0.145	1	9.86E-139
AXL	3.68E-141	-0.26809	0.015	0.237	7.36E-138
MKKS	1.38E-138	-0.2957	0.008	0.244	2.76E-135
MT1F	7.66E-133	0.525502	0.043	0.187	1.53E-129
CENPK	4.14E-132	0.335774	0.03	0.237	8.29E-129
HBB	1.17E-130	-0.52088	0.002	0.256	2.34E-127
SNCA	3.41E-128	-0.46497	0.005	0.256	6.83E-125
IRS2	7.91E-128	-0.36195	0.012	0.252	1.58E-124
CX3CR1	8.48E-09	-0.86955	0.002	0.557	1.70E-05
SSBP2	8.74E-126	-0.57792	0.003	0.26	1.75E-122
UCHL1	6.12E-124	-0.53892	0	0.263	1.22E-120
ARHGAP25	1.02E-121	0.377042	0.053	0.237	2.04E-118
NDRG2	1.46E-120	-0.4264	0.005	0.263	2.91E-117
CDKN1A	1.91E-119	-0.56342	0.013	0.244	3.82E-116
RASGRP1	8.18E-119	0.442336	0.039	0.794	1.64E-115
BBX	5.74E-118	0.374091	0.072	0.233	1.15E-114
RPA3	3.40E-117	0.49607	0.132	0.939	6.80E-114
HELLS	1.21E-116	0.293125	0.041	0.248	2.42E-113
UBE2C	2.20E-112	0.530015	0.09	0.233	4.41E-109
NUSAP1	4.00E-112	0.300028	0.057	0.813	8.00E-109
SPN	5.14E-111	0.478072	0.058	0.244	1.03E-107
TLR7	8.90E-111	-0.29169	0.004	0.221	1.78E-107
MILR1	6.99E-108	-0.33403	0.016	0.271	1.40E-104
ENC1	1.19E-107	-0.31381	0.009	0.275	2.38E-104
MCM3	3.03E-105	0.253674	0.055	0.802	6.06E-102
P2RY13	2.75E-07	-0.8674	0.008	0.557	0.00055
ANXA2	2.27E-104	0.44099	0.183	1	4.54E-101
OTUD1	1.24E-05	-0.84346	0.006	0.458	0.024869
CXorf21	4.08E-102	-0.31538	0.01	0.263	8.16E-99
ADRB2	1.34E-20	-0.82321	0.008	0.374	2.69E-17
TMIGD2	1.34E-100	-0.47415	0.002	0.286	2.68E-97
PDCD10	1.41E-99	0.341821	0.077	0.256	2.81E-96
FNIP2	4.81E-99	-0.27542	0.006	0.275	9.63E-96
PTAFR	6.60E-22	-0.79543	0.012	0.603	1.32E-18
AP1S2	1.71E-98	0.300421	0.072	0.26	3.42E-95
NLRP3	8.56E-98	-0.28472	0.005	0.252	1.71E-94
FSCN1	1.33E-11	-0.78568	0.011	0.576	2.65E-08
CEBPA	7.33E-96	-0.45745	0.01	0.275	1.47E-92
HSD17B14	9.87E-96	-0.35079	0.005	0.29	1.97E-92
LRRFIP1	2.14E-95	0.572049	0.12	0.885	4.28E-92
IL17RA	3.33E-95	-0.28577	0.008	0.286	6.66E-92
SLC9A3R1	4.69E-95	0.352511	0.043	0.275	9.37E-92
A2M	1.21E-16	-0.78557	0.019	0.599	2.43E-13
PFDN4	5.43E-94	0.28417	0.079	0.263	1.09E-90
DDX6	3.96E-92	0.286109	0.066	0.271	7.91E-89
NFIB	4.30E-91	-0.49085	0	0.298	8.60E-88
CST3	1.62E-26	-0.78157	0.439	0.973	3.24E-23
OLFML3	6.86E-15	-0.78025	0.003	0.58	1.37E-11
EGR2	1.17E-07	-0.77854	0.005	0.405	0.000233
QKI	2.47E-88	-0.26684	0.016	0.294	4.95E-85
TMEM106C	1.41E-87	0.300836	0.051	0.771	2.83E-84
TPM1	1.09E-85	-0.36328	0.015	0.298	2.18E-82
SLC31A2	2.55E-85	-0.2728	0.026	0.294	5.09E-82
GGCT	1.39E-84	0.262481	0.062	0.282	2.79E-81
MAFF	1.77E-84	-0.30133	0.007	0.252	3.53E-81
IFITM3	3.96E-82	0.435927	0.108	0.844	7.92E-79
SERPINA1	2.42E-81	-0.50181	0.079	0.794	4.84E-78
RNASE1	4.92E-46	-0.74156	0.026	0.351	9.84E-43
EGFL7	1.42E-79	-0.40585	0	0.302	2.83E-76
FCGR1B	6.62E-09	-0.71627	0.012	0.557	1.32E-05
ACY3	1.28E-24	-0.71138	0.004	0.385	2.56E-21
SPP1	1.10E-12	-0.70902	0.016	0.431	2.21E-09
ZNF331	7.42E-76	0.284418	0.075	0.786	1.48E-72
ADAM17	1.12E-75	0.335139	0.075	0.786	2.25E-72
QPRT	2.22E-75	-0.54864	0.009	0.313	4.44E-72
TOP1	4.52E-75	0.498603	0.096	0.29	9.04E-72
CARHSP1	1.26E-74	0.478258	0.103	0.29	2.52E-71
CCR1	5.43E-74	-0.25838	0.014	0.263	1.09E-70
ABCG2	8.91E-06	-0.70786	0	0.447	0.017817
LMNA	3.18E-72	0.26278	0.026	0.305	6.35E-69
CKS1B	4.68E-72	0.262069	0.067	0.302	9.35E-69
PTN	5.08E-157	-0.70593	0	0.233	1.02E-153
DUSP1	7.06E-33	-0.69028	0.358	0.962	1.41E-29
RIN2	3.15E-28	-0.68783	0.023	0.389	6.31E-25
TGIF1	3.30E-70	0.294718	0.048	0.309	6.60E-67
RHOC	1.39E-69	0.443375	0.068	0.305	2.78E-66
ADA	1.46E-69	0.319396	0.028	0.317	2.91E-66
GRPEL1	1.15E-68	0.298027	0.042	0.313	2.31E-65
HIST2H2BE	2.85E-68	-0.5642	0.003	0.324	5.70E-65
TLR1	2.93E-68	-0.38847	0.005	0.309	5.87E-65
S100A9	3.90E-68	0.434369	0.235	0.996	7.80E-65
ALOX5	6.12E-68	-0.26059	0.004	0.324	1.22E-64
UBE2T	3.87E-66	0.277879	0.042	0.317	7.73E-63
JUNB	5.95E-19	-0.68674	0.423	0.935	1.19E-15
DAB2	8.49E-19	-0.67583	0.018	0.374	1.70E-15
MS4A7	1.17E-45	-0.67268	0.024	0.347	2.33E-42
MKI67	3.90E-65	0.31344	0.05	0.317	7.79E-62
RNH1	1.47E-64	0.380126	0.079	0.313	2.93E-61
DHRS3	9.05E-12	-0.66809	0.018	0.435	1.81E-08
RAB32	1.96E-16	-0.65958	0.018	0.42	3.91E-13
TSC22D1	8.93E-63	-0.3396	0.016	0.328	1.79E-59
SAT1	1.40E-17	-0.65436	0.57	0.958	2.80E-14
KIF20B	1.54E-59	0.271291	0.039	0.328	3.08E-56
CKB	1.34E-25	-0.64332	0.014	0.618	2.68E-22
NAGA	1.01E-58	-0.38728	0.013	0.328	2.02E-55
SFMBT2	1.08E-58	-0.47938	0.013	0.317	2.16E-55
RHBDF2	1.26E-58	-0.36698	0.019	0.332	2.52E-55
NUCB2	2.43E-58	0.419403	0.069	0.748	4.86E-55
S100A1	4.11E-270	-0.61625	0.002	0.145	8.22E-267
CTSB	5.99E-56	-0.30069	0.232	0.95	1.20E-52
MEF2C	3.03E-09	-0.61558	0.027	0.58	6.06E-06
SLF1	5.69E-53	0.316137	0.038	0.34	1.14E-49
CCND1	3.95E-52	-0.5756	0.004	0.347	7.89E-49
SLA	6.19E-52	0.443016	0.115	0.336	1.24E-48
LSM4	6.55E-52	0.474495	0.111	0.336	1.31E-48
POU2F2	7.51E-52	0.41851	0.098	0.336	1.50E-48
SH3TC1	5.86E-51	0.4739	0.075	0.34	1.17E-47
IFI30	1.47E-50	0.540519	0.266	1	2.94E-47
MCL1	2.51E-50	-0.28475	0.123	0.798	5.01E-47
DNAJC9	7.72E-50	0.281869	0.051	0.344	1.54E-46
SPRY1	1.97E-104	-0.59693	0.005	0.279	3.94E-101
CENPH	7.97E-49	0.261125	0.043	0.698	1.59E-45
BCL2A1	1.03E-48	0.420906	0.076	0.344	2.05E-45
CSF1R	1.13E-48	-0.43418	0.044	0.698	2.26E-45
NASP	1.64E-48	0.448827	0.128	0.344	3.28E-45
DNAJB1	1.78E-20	-0.59351	0.109	0.691	3.56E-17
CMTM3	1.94E-47	0.258029	0.063	0.347	3.87E-44
CSF3R	8.52E-11	-0.59347	0.006	0.424	1.70E-07
ITGAM	1.88E-46	-0.32791	0.007	0.347	3.76E-43
ATP1B1	3.45E-41	-0.59157	0.021	0.363	6.90E-38
RGS2	2.22E-22	-0.58979	0.19	0.775	4.43E-19
AFMID	1.97E-45	0.257182	0.024	0.355	3.93E-42
ATF3	1.40E-44	0.268805	0.039	0.355	2.81E-41
RPS20	1.12E-13	0.586595	0.858	0.939	2.25E-10
H2AFV	1.71E-31	0.588666	0.184	0.397	3.42E-28
SLC25A37	2.05E-42	-0.37463	0.039	0.359	4.10E-39
CCAR1	2.11E-42	0.348537	0.047	0.359	4.22E-39
ATAD2	6.76E-42	0.328383	0.032	0.672	1.35E-38
SOCS6	7.35E-42	-0.46165	0.003	0.347	1.47E-38
HHEX	1.37E-41	-0.30842	0.018	0.363	2.74E-38
DUSP4	1.47E-239	0.58922	0.082	0.996	2.94E-236
HLA-DMA	4.33E-41	0.281301	0.288	1	8.66E-38
DHRS9	6.30E-41	-0.37454	0.029	0.363	1.26E-37
IGF1	1.61E-40	-0.56032	0.006	0.363	3.21E-37
CTNND1	2.77E-40	-0.3214	0.006	0.366	5.54E-37
TMEM219	5.40E-40	0.30065	0.086	0.363	1.08E-36
CD83	2.86E-39	-0.31231	0.053	0.691	5.71E-36
YBX3	4.98E-39	-0.4024	0.028	0.366	9.95E-36
ATP2A3	1.11E-38	0.281658	0.023	0.195	2.22E-35
MARCKS	2.69E-38	-0.43934	0.069	0.706	5.39E-35
WIPI1	1.07E-36	-0.27234	0.007	0.363	2.13E-33
VSIG4	1.33E-36	-0.45486	0.107	0.744	2.65E-33
TRIB1	9.16E-36	-0.53235	0.012	0.359	1.83E-32
SNX5	2.86E-35	0.263435	0.064	0.374	5.71E-32
IFNGR1	7.66E-35	-0.50321	0.08	0.71	1.53E-31
TNF	1.10E-34	-0.37026	0.011	0.302	2.19E-31
CD55	8.11E-34	0.591513	0.071	0.378	1.62E-30
IQGAP1	1.67E-210	0.597835	0.091	0.985	3.35E-207
ZFHX3	9.08E-34	-0.44422	0.006	0.336	1.82E-30
TUBB4B	6.80E-33	0.266244	0.099	0.382	1.36E-29
ANP32B	7.06E-15	0.59877	0.221	0.485	1.41E-11
IER2	2.68E-32	-0.40216	0.144	0.771	5.35E-29
ICAM1	2.87E-32	-0.33694	0.021	0.355	5.74E-29
LYL1	7.67E-32	-0.33612	0.015	0.382	1.53E-28
TAGLN2	1.05E-100	0.600271	0.187	1	2.10E-97
GIMAP4	2.09E-31	0.31496	0.152	0.782	4.18E-28
FERMT3	4.32E-31	0.541815	0.079	0.385	8.65E-28
KLF4	6.25E-31	-0.4473	0.032	0.363	1.25E-27
CTHRC1	3.51E-30	0.364547	0.028	0.187	7.03E-27
CDK2AP2	2.02E-90	0.606957	0.088	0.267	4.05E-87
CYBB	8.45E-30	-0.49343	0.101	0.71	1.69E-26
FXYD5	5.69E-63	0.607664	0.235	0.989	1.14E-59
HSPA6	1.20E-28	-0.29613	0.013	0.328	2.39E-25
BTG2	1.78E-28	-0.53456	0.075	0.687	3.57E-25
IRF1	1.10E-10	0.609484	0.099	0.462	2.20E-07
BUB3	4.82E-28	0.501025	0.128	0.401	9.63E-25
FOLR2	1.41E-27	-0.29786	0.047	0.656	2.83E-24
PCSK7	3.68E-30	0.610607	0.089	0.389	7.36E-27
RAD21	6.62E-27	0.437749	0.078	0.397	1.32E-23
TAGAP	6.96E-27	-0.53086	0.049	0.653	1.39E-23
MAP1B	1.12E-26	-0.537	0.001	0.393	2.23E-23
HOPX	9.22E-225	0.61598	0.087	0.992	1.84E-221
AIF1	3.98E-26	-0.40966	0.33	0.95	7.97E-23
BATF	1.50E-183	0.636263	0.115	1	3.01E-180
RPL17	6.62E-26	0.463958	0.976	1	1.32E-22
RGCC	5.00E-49	0.636931	0.072	0.344	1.00E-45
NAAA	1.18E-24	0.298252	0.031	0.397	2.37E-21
ANXA1	7.98E-165	0.648431	0.125	0.996	1.60E-161
PTPN7	4.65E-216	0.659071	0.095	1	9.30E-213
NRP2	1.84E-24	-0.46182	0.009	0.389	3.68E-21
MAD2L1	2.18E-24	0.37801	0.053	0.653	4.37E-21
LITAF	2.26E-24	0.583309	0.141	0.416	4.51E-21
PTPRC	7.69E-24	0.487112	0.347	1	1.54E-20
EPSTI1	1.34E-23	0.493313	0.061	0.405	2.68E-20
GNB4	2.20E-23	-0.25601	0.012	0.401	4.41E-20
FTH1	4.54E-23	-0.55999	0.947	0.989	9.07E-20
PLIN2	7.14E-23	0.32426	0.075	0.408	1.43E-19
C3AR1	1.06E-22	-0.47954	0.045	0.641	2.11E-19
TLR10	1.29E-22	-0.52454	0.009	0.401	2.57E-19
RPLP1	1.49E-22	0.384943	1	1	2.99E-19
FCER1G	2.11E-22	-0.51761	0.207	0.794	4.22E-19
DEK	4.60E-14	0.659472	0.18	0.473	9.20E-11
JUN	3.18E-22	-0.44757	0.187	0.779	6.35E-19
ANP32E	1.79E-19	0.668433	0.11	0.427	3.57E-16
TUBA1A	6.62E-22	0.299892	0.062	0.656	1.32E-18
C1orf61	1.19E-21	-0.44643	0.003	0.405	2.37E-18
EIF1B	1.31E-21	0.310069	0.097	0.416	2.62E-18
ADAM28	2.16E-21	-0.55265	0.013	0.553	4.33E-18
HIST1H4C	2.89E-57	0.674257	0.183	0.321	5.78E-54
ANXA6	2.22E-89	0.676974	0.107	0.263	4.45E-86
EMP3	1.76E-94	0.680461	0.194	1	3.51E-91
TYROBP	2.31E-20	-0.52358	0.372	0.95	4.63E-17
GADD45B	7.92E-20	-0.53412	0.105	0.687	1.58E-16
RPS27L	1.20E-19	0.579424	0.266	0.87	2.41E-16
SERPINB9	1.25E-19	0.25749	0.079	0.42	2.50E-16
RPL5	4.60E-19	0.686509	0.883	0.954	9.20E-16
HLA-DPB1	9.81E-10	0.687484	0.646	1	1.96E-06
CTNNB1	2.71E-54	0.691842	0.257	1	5.42E-51
DDIT4	7.56E-80	0.697345	0.209	0.992	1.51E-76
AHNAK	1.02E-18	0.527036	0.043	0.233	2.04E-15
C16orf74	1.11E-18	0.278182	0.02	0.134	2.21E-15
LILRB4	1.21E-18	-0.37315	0.041	0.626	2.42E-15
PLD3	1.80E-18	-0.28325	0.08	0.66	3.60E-15
ITGB2	2.29E-18	0.272446	0.249	0.836	4.59E-15
MTHFD2	1.07E-09	0.698148	0.117	0.473	2.13E-06
STMN1	2.49E-08	0.700505	0.267	0.798	4.97E-05
TM6SF1	2.15E-17	-0.36596	0.006	0.412	4.31E-14
RPS2	3.32E-17	0.373697	0.99	1	6.64E-14
IFITM2	1.03E-46	0.725121	0.276	1	2.06E-43
C2	8.03E-17	-0.56934	0.023	0.412	1.61E-13
CD8A	8.91E-213	0.726032	0.097	1	1.78E-209
GMFG	1.86E-16	0.404556	0.385	1	3.73E-13
KLRD1	8.05E-160	0.743047	0.131	1	1.61E-156
GYG1	3.68E-16	0.412084	0.07	0.645	7.36E-13
GNG7	5.68E-16	-0.44768	0.002	0.412	1.14E-12
RGS10	6.61E-16	0.466095	0.191	0.763	1.32E-12
CEBPD	6.88E-16	-0.34349	0.044	0.424	1.38E-12
ZFP36	8.09E-16	-0.33652	0.21	0.771	1.62E-12
MFNG	8.61E-16	0.301142	0.05	0.427	1.72E-12
IER3	1.34E-15	-0.53446	0.119	0.683	2.68E-12
RPLP0	1.53E-15	0.519962	0.94	1	3.05E-12
FTL	1.77E-15	-0.51563	0.967	1	3.55E-12
RPS6	1.91E-15	0.361088	0.997	1	3.82E-12
HCST	1.92E-08	0.745433	0.426	1	3.85E-05
TRAF3IP3	7.83E-180	0.762333	0.102	0.141	1.57E-176
LGALS3	9.74E-15	0.356459	0.129	0.45	1.95E-11
DUSP6	9.84E-15	-0.57815	0.02	0.42	1.97E-11
LCP1	1.63E-60	0.779313	0.227	0.973	3.26E-57
ARID5B	1.01E-76	0.786126	0.084	0.802	2.03E-73
EEF1D	4.73E-12	0.791044	0.561	0.767	9.45E-09
RPL36A	3.25E-14	0.32622	0.926	1	6.50E-11
NUCKS1	9.00E-11	0.79846	0.201	0.5	1.80E-07
RPS26	1.03E-13	0.277233	0.916	1	2.05E-10
TXNIP	2.09E-29	0.821898	0.27	0.924	4.19E-26
PTTG1	1.07E-127	0.846445	0.159	1	2.14E-124
SDCBP	1.79E-13	-0.29324	0.151	0.702	3.58E-10
PFN1	2.23E-13	0.306536	0.885	0.977	4.47E-10
TRIM59	3.39E-13	0.348903	0.037	0.172	6.78E-10
IGSF6	5.97E-13	-0.39753	0.073	0.63	1.19E-09
S100A8	1.76E-14	0.865235	0.122	0.439	3.52E-11
AC058791.1	2.37E-210	0.86979	0.099	1	4.74E-207
CD53	4.10E-12	0.281058	0.248	0.802	8.20E-09
PRDM1	5.12E-66	0.900115	0.236	1	1.02E-62
LGALS1	4.56E-34	0.908717	0.357	0.427	9.12E-31
S100A10	1.28E-70	0.915661	0.226	1	2.56E-67
MT-CO2	2.04E-11	0.30526	0.992	1	4.08E-08
GAS6	2.86E-11	-0.31959	0.009	0.435	5.72E-08
RUNX1	3.52E-11	-0.37441	0.016	0.435	7.04E-08
TMEM107	4.10E-11	-0.28994	0.044	0.443	8.19E-08
FOXP1	3.02E-155	0.917182	0.132	0.996	6.05E-152
IL7R	4.46E-145	0.932014	0.143	1	8.91E-142
PDLIM1	9.17E-168	0.940959	0.125	1	1.83E-164
SRI	1.52E-10	0.41281	0.171	0.489	3.05E-07
TSC22D3	2.31E-10	0.264004	0.362	0.908	4.62E-07
IPCEF1	2.33E-10	-0.40488	0.016	0.42	4.65E-07
IRF8	3.07E-10	-0.37316	0.046	0.599	6.14E-07
ANXA5	3.39E-10	0.312722	0.161	0.489	6.78E-07
SPINT2	3.42E-10	-0.52703	0.026	0.443	6.83E-07
SERPINB1	4.42E-10	0.365271	0.105	0.466	8.85E-07
SLC25A5	2.66E-12	0.982428	0.496	0.698	5.33E-09
RAC2	2.29E-65	1.055226	0.236	1	4.59E-62
FXYD2	1.66E-42	1.069118	0.28	1	3.33E-39
TREM2	1.29E-09	-0.31872	0.005	0.565	2.58E-06
SHTN1	1.69E-09	-0.49865	0.014	0.443	3.38E-06
MT-ND3	1.81E-09	0.473159	0.859	0.981	3.63E-06
S100A4	1.16E-13	1.114301	0.674	1	2.32E-10
LPAR6	3.65E-09	-0.57893	0.022	0.576	7.30E-06
GAPDH	3.66E-09	0.360837	0.927	1	7.32E-06
HMGB1	8.28E-18	1.115085	0.627	0.824	1.66E-14
RPL22L1	8.43E-09	0.538712	0.163	0.695	1.69E-05
PTGDS	8.57E-97	1.239914	0.19	1	1.71E-93
ADORA3	1.48E-08	-0.41422	0.009	0.546	2.97E-05
ITM2A	7.52E-103	1.252947	0.183	1	1.50E-99
TRBC2	1.28E-70	1.291133	0.226	1	2.56E-67
SLC7A7	7.95E-08	-0.46096	0.037	0.45	0.000159
MT-ND1	8.79E-08	0.522267	0.803	0.977	0.000176
CMC1	2.43E-27	1.295633	0.323	1	4.85E-24
TRAC	1.44E-07	1.361453	0.409	1	0.000287
MT-ND2	1.76E-07	0.328704	0.937	1	0.000352
CHCHD10	2.50E-07	0.335493	0.202	0.523	0.000499
CD79A	1.88E-76	1.452771	0.218	1	3.77E-73
SLC16A3	2.83E-07	-0.43218	0.029	0.565	0.000566
EGR1	3.64E-07	-0.38136	0.044	0.584	0.000727
H1F0	4.30E-07	-0.43014	0.004	0.45	0.00086
RPL7A	4.81E-07	0.385761	0.876	0.989	0.000962
EVI2B	7.48E-07	0.363181	0.133	0.496	0.001497
C1orf131	1.26E-06	0.348345	0.048	0.584	0.002514
SKA2	1.53E-06	0.319802	0.078	0.607	0.003054
VIM	1.55E-06	0.548418	0.427	0.969	0.003102
TRBC1	4.84E-72	1.489243	0.224	1	9.68E-69
LTB	4.01E-26	1.823151	0.315	0.985	8.02E-23
RGS19	1.31E-05	-0.43809	0.057	0.584	0.026262
GPR183	1.42E-05	-0.40339	0.099	0.489	0.028442
LGMN	2.37E-05	-0.48599	0.038	0.569	0.047404
NPL	2.52E-05	-0.52144	0.026	0.466	0.050337
TMIGD3	2.77E-05	-0.48611	0.012	0.462	0.055331
HSPA1B	2.81E-05	-1.25059	0.055	0.576	0.0563
S100A11	2.92E-05	-0.57663	0.396	0.821	0.058485
NUPR1	2.95E-05	-0.33696	0.03	0.466	0.059012
CXCR4	3.23E-05	1.093525	0.613	1	0.064537
PDK4	3.35E-05	-1.07695	0.012	0.55	0.066911
BHLHE41	4.36E-05	-0.84945	0.013	0.55	0.087294
HLA-DRB1	5.41E-05	-0.35041	0.742	1	0.108272
HMGN2	5.57E-05	0.542279	0.342	0.847	0.111311
DUT	6.89E-05	0.592744	0.179	0.534	0.137793
SLCO2B1	8.59E-05	-0.31448	0.014	0.45	0.171702
LPAR5	0.000128	-0.48452	0.006	0.462	0.255606
NR4A1	0.000144	-0.28851	0.016	0.462	0.287745
RHOB	0.000148	-0.84836	0.017	0.55	0.295977
CSF2RA	0.000244	-0.55898	0.025	0.55	0.487616
SPI1	0.000246	-0.29657	0.069	0.584	0.49193
HSPB1	0.000269	-0.28355	0.139	0.511	0.537404
CEBPB	0.000327	-0.32416	0.064	0.58	0.654669
HNRNPH1	0.00033	0.760791	0.468	1	0.659242
INPP5D	0.00035	-0.43356	0.032	0.557	0.699692
GPR34	0.000359	-0.44802	0.015	0.546	0.717113
HMGB2	0.000359	1.311171	0.451	0.996	0.71738
ITM2C	0.000378	-0.38123	0.021	0.55	0.756766
PIK3IP1	0.000381	0.279222	0.074	0.492	0.762633
LDHA	0.000408	0.559444	0.303	0.618	0.816764
FCGR3A	0.000416	-0.4745	0.063	0.576	0.83165
TBXAS1	0.000554	-0.42683	0.022	0.466	1
OLR1	0.000763	-0.62195	0.008	0.458	1
CCL5	0.000797	0.437249	0.634	1	1
TUBA1B	0.00084	0.779423	0.549	0.882	1
APOC1	0.001233	-0.62958	0.303	0.721	1
ZFP36L2	0.001538	0.337145	0.389	0.859	1
FOSB	0.001875	-0.55043	0.058	0.569	1
CSF1	0.002307	-0.57872	0.005	0.466	1
C12orf45	0.002807	0.337351	0.048	0.489	1
LTC4S	0.003435	-0.72142	0.02	0.542	1
SERPINF1	0.003714	-0.30305	0.052	0.561	1
CYBA	0.003845	0.525643	0.543	0.901	1
HTRA1	0.003884	-0.65628	0.003	0.531	1
CD63	0.004153	0.36458	0.321	0.775	1
ARHGDIB	0.004482	0.250719	0.531	0.821	1
TPI1	0.005605	0.709065	0.452	0.79	1
EZR	0.006022	0.320487	0.116	0.523	1
NFKBID	0.008314	-0.31397	0.028	0.542	1
HNRNPA2B1	0.008975	0.637667	0.432	0.752	1
COTL1	0.011432	0.808302	0.486	0.828	1
KLF2	0.011787	-0.35417	0.039	0.55	1
FCGR2A	0.011833	-0.33268	0.021	0.534	1
NBL1	0.012004	-0.62458	0.006	0.477	1
PPT1	0.016939	0.334068	0.075	0.573	1
TUBB	0.025557	1.060503	0.406	0.905	1
NCL	0.029692	0.760466	0.306	0.767	1
CLIC1	0.029748	0.737911	0.411	0.752	1
TLR4	0.030913	-0.50283	0.011	0.481	1
CSRNP1	0.032545	-0.3615	0.015	0.473	1
EEF1B2	0.035499	0.407861	0.664	0.924	1
VAMP8	0.039628	0.311765	0.267	0.626	1
LAT2	0.054515	-0.48467	0.028	0.492	1
PHACTR1	0.057219	-0.80759	0.037	0.496	1
IFI16	0.065228	0.608125	0.219	0.603	1
CYSLTR1	0.078568	-0.6272	0.019	0.519	1
ELF1	0.090847	0.326417	0.135	0.603	1
HLA-E	0.096029	0.442311	0.439	0.897	1
REST	0.127454	0.293489	0.042	0.504	1
MAF	0.169999	-0.31342	0.014	0.492	1
CXCL16	0.173623	-0.56187	0.031	0.496	1
MKNK1	0.204109	-0.45756	0.029	0.5	1
CH25H	0.216392	-1.13684	0.003	0.454	1
DBI	0.219191	0.566369	0.315	0.679	1
HBA2	0.272516	-1.85234	0.001	0.511	1
FPR1	0.279672	-0.47313	0.015	0.519	1
HLA-DPA1	0.358735	0.533764	0.556	1	1
ADAP2	0.392723	-0.43015	0.013	0.473	1
PLTP	0.41267	-0.37978	0.042	0.531	1
HSP90AB1	0.462418	0.449422	0.525	0.893	1
ARL6IP1	0.485381	0.302649	0.166	0.607	1
RNASE6	0.514019	-0.26725	0.052	0.534	1
LAPTM5	0.518558	0.591489	0.45	0.859	1
GNG5	0.520191	0.604291	0.221	0.622	1
PKM	0.520607	0.594787	0.334	0.744	1
MYL12A	0.565611	0.657066	0.56	1	1
CD37	0.574315	0.424605	0.371	0.782	1
CD86	0.630939	-0.43594	0.026	0.508	1
HLA-DRA	0.658756	-0.32653	0.887	1	1
CD99	0.689583	0.33758	0.237	0.641	1
SNRPB	0.696812	0.371837	0.259	0.653	1
PLA2G7	0.739163	-0.68384	0.024	0.511	1
S100A6	0.761151	1.523916	0.509	1	1
CORO1A	0.768598	0.38342	0.587	1	1
CRIP1	0.820923	1.601983	0.497	1	1
CITED2	0.841241	-0.85163	0.068	0.531	1
HIST1H1C	0.892343	-0.44228	0.026	0.511	1
NME2	0.983861	0.703667	0.532	1	1

## Discussion

CNS tissue is the key site of PCNSL pathogenesis and progression which consists of diverse types of cells. Understanding the molecular phenotype of each cell type to the PCNSL is an important step to reveal the pathogenesis of PCNSL. The availability of scRNA-seq data allows us to perform molecular and functional heterogeneity analysis for different cells in the PCNSL tissue. In this study, we investigated CNS tissues at a single-cell resolution using gene expression profiling. We mapped the immune microenvironment of PCNSL and identified the cellular markers and functional signals of immune cells such as B cells, macrophages, and dendritic cells in PCNSL, which provided a partial basis for guiding the precise treatment of PCNSL.

Abnormal proliferation and differentiation of B cells are directly related to the pathological basis of PCNSL, and scRNA-seq enables us to directly parse the heterogeneity of B cells from single-cell resolution. We identified that B cells can be divided into three subgroups with completely different gene expression and function, including a small number of terminally differentiated plasma cells (IGHG1, IGHG3, IGHG4). We identified that B cells can be divided into three subgroups with completely different gene expression and function, including a small number of terminally differentiated plasma cells. In addition to the subsets of normal B cells (B cell-1 overexpressed CD79A and CD79B), B cell-2 showed an important role in antigen processing and regulating the cytotoxicity of T cells, and its function was similar to that of dendritic cells, which may be related to the abnormal differentiation of B cells in PCNSL. This also indicated from the molecular phenotype that B cells in PCNSL were in the pathological overactivation state.

Cell–cell communication between B cells and other immune cells is also an important mechanism for the progression of PCNSL disease (12, 15, 33). Surprisingly, we found that CD74 might be a key target to regulate the communication between B cells and the other three types of immune cells (T cell, macrophage, dendritic cell) and CD74–MIF was identified to be the most significant interaction. The macrophage migration inhibitory factor (MIF) receptor CD74 is overexpressed in various neoplasms, mainly in hematologic tumors, and currently investigated in clinical studies. Zeiner et al. summarized that the MIF–CD74A interaction is restricted to macrophages, associated with survival in glioma (34). CD74 is quickly internalized and recycles after antibody binding; therefore, it constitutes an attractive target for antibody-based treatment strategies. CD74 has been further described as one of the most upregulated molecules in human glioblastomas. They also pointed that CD74 expression in human gliomas is restricted to GAMs and positively associated with patient survival. Moreover, CD74 represents a positive prognostic marker most probably because of its association with an M1-polarized immune milieu in high-grade gliomas. Our study strongly suggests that CD74 of B cells in PCNSL may be an important cellular and molecular pathological basis for its coordination and disturbance with other immune cells to induce the disorder of the immune microenvironment. This may provide an important basis for targeted therapy of PCNSL. However, there are still limitations in the above conclusions, namely, that malignant and non-malignant B cells in PCNSL have not been identified and distinguished. These analyses are mainly based on the mixed population of B cells in PCNSL. In this regard, the separated malignant and non-malignant B cells from PCNSL tumors should be accurately analyzed for the tumor cell heterogeneity of PCNSL in the future.

In addition, we also demonstrated that immune cells would be affected by the tumor microenvironment. Heterogeneities were also observed in these cell types. We identified T cell, T helper cell, NKT cell, and MPC subtypes within T cell populations that possessed different ontologies. Moreover, we also identified three subtypes within the dendritic cells, including conventional dendritic cell, myeloid dendritic cell, and plasmacytoid dendritic cell. They also enriched into NF-κB signaling, antigen processing and presentation, and neutrophil degranulation pathways, respectively. Further studies on the immune regulation mechanism of these subtypes of T cells and dendritic cells might provide a new strategy for the treatment of PCNSL.

In conclusion, our single-cell analysis enabled us to study the cellular heterogeneity and gene expression characteristics of PCNSL at a single-cell resolution and whole-transcriptome scale. Our study is the first to analyze the cellular and molecular pathological map at single-cell resolution. Despite the small size of single-cell samples in our study, it reveals novel potential for targeted immunotherapy of PCNSL.

## Data Availability Statement

The raw and processed data supporting the findings in this study are available from the corresponding author, and has been submitted to the Gene Expression Omnibus (GEO) database under the accession code GSE181304 (https://www.ncbi.nlm.nih.gov/geo/query/acc.cgi?acc=GSE181304).

## Ethics Statement

Written informed consent was not obtained from the individual(s) for the publication of any potentially identifiable images or data included in this article.

## Author Contributions

Conceptualization, B.Y.W. and Z.L.; Methodology, B.Y.W. and Z.L.; Validation, Y.F. and G.C.; Formal Analysis, B.Y.W. and Z.L.; Resources, B.Y.W., S.W.W., C.D., W.R. and F.Y.; Data Curation, Y.F.; Visualization, Y.F.; Writing-Original Draft, B.Y.W., Z. L. and Y.F.; Writing-Review & Editing, B.Y.W., Z.L, and J.N.Z.; Supervision, G.C., and J.N.Z. All authors contributed to the article and approved the submitted version.

## Funding

This study was funded by the Innovation Cultivation Fund of the Sixth Medical Center of PLA General Hospital (CXPY201913) and the Youth Science Foundation Project of the National Natural Science Foundation of China (81801165).

## Conflict of Interest

The authors declare that the research was conducted in the absence of any commercial or financial relationships that could be construed as a potential conflict of interest.

## Publisher’s Note

All claims expressed in this article are solely those of the authors and do not necessarily represent those of their affiliated organizations, or those of the publisher, the editors and the reviewers. Any product that may be evaluated in this article, or claim that may be made by its manufacturer, is not guaranteed or endorsed by the publisher.
